# Role of Substitution Patterns in Four Regioisomeric Tetraphenylethylene–Thiophene Derivatives

**DOI:** 10.3390/molecules30142953

**Published:** 2025-07-13

**Authors:** Shuai Hou, Hanxiao Tian, Ruiyao Li, Zishuai Huang, Dongyuan Zhu, Fan Xiao, Yunmeng Zhao, Jingjing Xu

**Affiliations:** 1 The Education Ministry Key Lab of Resource Chemistry, Shanghai Key Laboratory of Rare Earth Functional Materials, Shanghai Frontiers Science Center of Biomimetic Catalysis, College of Chemistry and Materials Science, Shanghai Normal University, Shanghai 200234, China; h1026712305@163.com (S.H.); thx2218@163.com (H.T.); 1000549716@smail.shnu.edu.cn (R.L.); 13782223290@163.com (Z.H.); 13619559574@163.com (D.Z.); 1000502140@smail.shnu.edu.cn (F.X.); 2 Key Laboratory of Smart Manufacturing in Energy Chemical Process, Ministry of Education, East China University of Science and Technology, Shanghai 200237, China

**Keywords:** aggregation-induced emission, tetraphenylethylene, thiophene, PLQY, substitution pattern

## Abstract

Tetraphenylethylene (TPE)–thiophene compounds are promising candidates for stimuli-responsive luminescent materials, yet systematic investigations into the influence of substitution patterns on their photophysical properties remain limited. Herein, four regioisomeric TPE–thiophene derivatives have been synthesized by systematically varying the number and positions of TPE substituents on the thiophene core. A comprehensive spectroscopic characterization reveals that substitution patterns critically modulate the photoluminescence quantum yields (PLQYs). The ortho-monosubstituted isomer exhibits the highest PLQY (52.86% in solid state) compared with the meta-monosubstituted isomer (13.87% in solid state). Interestingly, thiophenes with two or three TPEs substituted at positions 2,5 or 2,3,5 have lower PLQYs, which is rare due to the common understanding that increasing the number of AIE parts should increase the PLQY. Further single-crystal structure analyses show that the key factor impacting the PLQY is the dihedral angles of the TPE subunit, which determines the degree of intramolecular twisting. This work establishes regiochemistry as a powerful design lever for tuning TPE–thiophene photophysics, offering underlying principles for the design of TPE-based thiophene molecules with high photoluminescent performance in the future.

## 1. Introduction

In recent years, thiophene derivatives have attracted extensive attention in the field of organic optoelectronic materials (e.g., organic light-emitting diodes (OLEDs) [1,2,3,4], electrochemical energy storage devices [5,6,7,8,9], and electrochromic devices) [10,11,12,13] due to their conjugated backbone structures and excellent charge transport capabilities [14]. For instance, Ozturk et al. systematically investigated the synthesis and properties of sulfur-containing fused aromatic hydrocarbons, revealing their potential for high charge mobility in polymeric material [15]. Meanwhile, Zhu et al. demonstrated the promising capacitive properties of polythienothiophene films prepared via electrochemical polymerization, highlighting their applications in energy storage [16].

Tetraphenylethylene (TPE), a classic aggregation-induced emission (AIE) moiety [17,18,19,20,21,22], has become a critical strategy for improving the optical performance of organic materials due to its non-planar conformation and high solid-state fluorescence quantum efficiency [1,16]. The introduction of TPE effectively avoids the fluorescence quenching caused by π-π stacking in aggregated states [5,23]. Recent studies have attempted to integrate TPE with thiophene-based skeletons to optimize optoelectronic copolymers, achieving a significant enhancement in electrochromic efficiency [16,24] (up to 309 cm^2^/C). Ozturk et al. further proposed incorporating TPE units at the periphery of thiophene backbones, realizing blue-emitting OLED devices (with an EQE of 2.45%) and elucidating the critical role of molecular non-planarity in device stability [25].

**Scheme 1:**
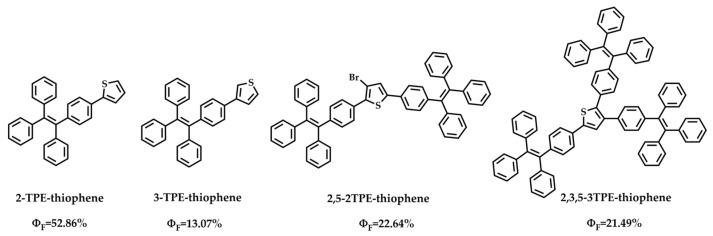
The structures and photoluminescence quantum yields of four TPE–thiophene derivatives in solid states (Φ_F_ = photoluminescence quantum yield).

However, there is little research focusing on comprehensively investigating the impact of substitution patterns and the numbers of TPE parts on photophysical properties of the TPE–thiophene compound. The only related research is from Lu’s group, which studied the effect of different binding sites of two TPE parts on a thiophene core in 2017 [10]. Yet there is no research on the effect of TPE numbers and mono-substituted isomers on these kinds of compounds. To better understand the effect of TPE parts on the thiophene core, we designed four regioisomeric TPE–thiophene derivatives by systematically varying the number and positions of TPE substituents on the thiophene core (Scheme 1). The result showed that the ortho-monosubstituted isomer 2-TPE-thiophene exhibited the highest photoluminescence quantum yields (PLQYs), while the thiophene with two/three TPE substituted at position 2,5/2,3,5 (2,5-2TPE-thiophene/2,3,5-3TPE-thiophene) had the lowest PLQY. We investigated the underlying factors responsible for the differences in photophysical properties among these regioisomers using detailed single-crystal analyses.

## 2. Results and Discussion

### 2.1. Synthesis and Characteristics

**Scheme 2:**
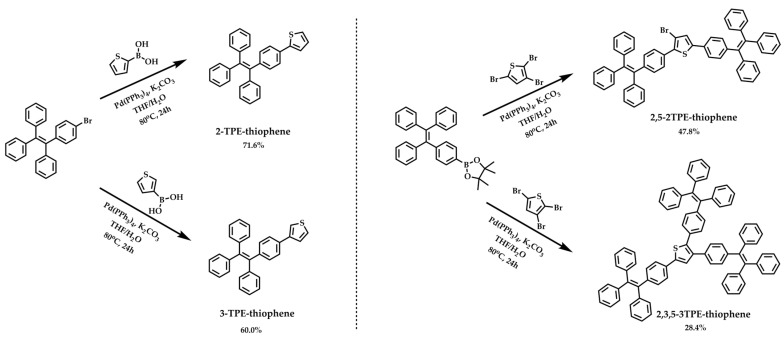
Synthetic routes of four TPE–thiophene derivatives.

Four TPE–thiophene derivatives (2-TPE-thiophene, 3-TPE-thiophene, 2,5-2TPE-thiophene and 2,3,5-3TPE-thiophene) were synthesized through straightforward Suzuki couplings using 1(4-bromophenyl)-1,2,2-triphenylethylene with thiophene-2-boronic acid and thiophene-3-boronic acid or 4,4,5,5-tetramethyl-2-(4-(1,2,2-triphenylvinyl)phenyl)-1,3,2-dioxaborolane with 2,3,5-Tribromothiophene using different ratios (Scheme 2).

^1^H NMR, ^13^C NMR, and Mass Spectroscopy were used to verify the structure of 2-TPE-thiophene and 3-TPE-thiophene (Figures S1, S2, S5, S6, S9, and S10). The synthesis yields were both above 60%. ^1^H NMR, ^13^C NMR, and mass spectroscopy were used to verify the structure of 2,5-2TPE-thiophene and 2,3,5-3TPE-thiophene (Figures S3, S4, S7, S8, S11, and S12), which showed lower yields than 2-TPE-thiophene and 3-TPE-thiophene.

### 2.2. AIE Properties in Solution

To study their optical properties, UV-Vis absorption spectra and fluorescence spectra were performed. The detailed data are listed in Table 1 and Figures S20–S23. Mono-substituted isomers 2-TPE-thiophene and 3-TPE-thiophene show similar maximum absorbance peaks at around 325 nm in THF solution. With more TPE parts induced, the extended conjugation would cause an absorption spectrum redshift from 321 nm to 365 nm.

**Figure 1:**
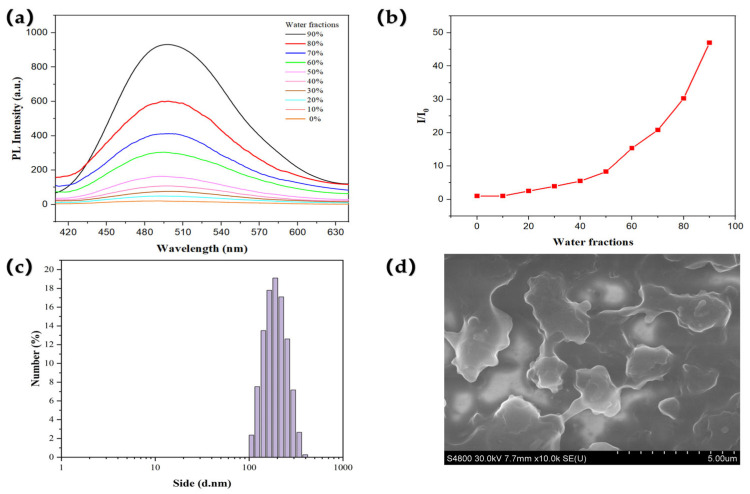
(**a**) Emission spectra of 2-TPE-thiophene in THF/H_2_O mixtures (concentration = 10 μM; λ_ex_ = 331 nm). (**b**) Plots of the emission intensities of 2-TPE-thiophene in THF/H_2_O mixtures versus water fractions (f_w_ = 0) at 498 nm. (**c**) The DLS plot of 2-TPE-thiophene in 90% (vol%) H_2_O/THF solution. (**d**) SEM of 2-TPE-thiophene in 90% (vol%) H_2_O/THF solution.

**Figure 2:**
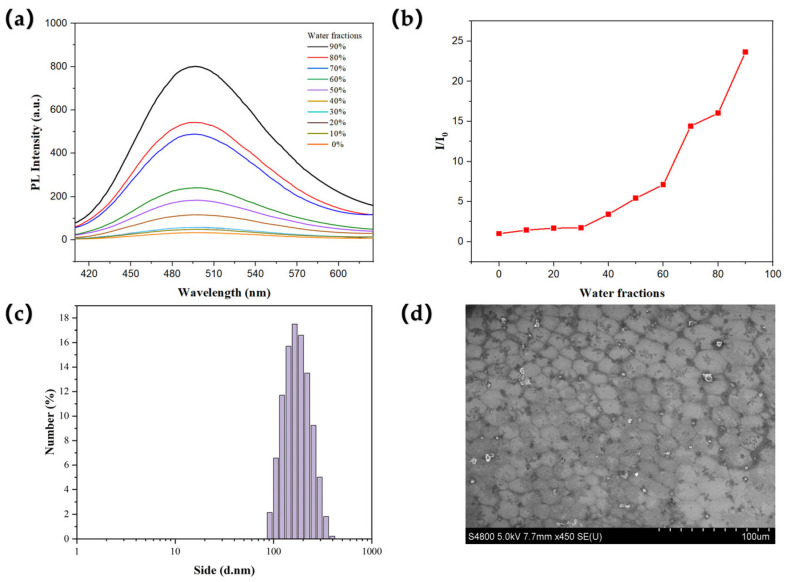
(**a**) Emission spectra of 3-TPE-thiophene in THF/H_2_O mixtures (concentration = 10 μM. λ_ex_ = 321 nm). (**b**) Plots of the emission intensities of 3-TPE-thiophene in THF/H_2_O mixtures versus water fractions (f_w_ = 0) at 498 nm. (**c**) The DLS plot of 3-TPE-thiophene in 90% (vol%) H_2_O/THF solution. (**d**) SEM of 3-TPE-thiophene in 90% (vol%) H_2_O/THF solution.

**Figure 3:**
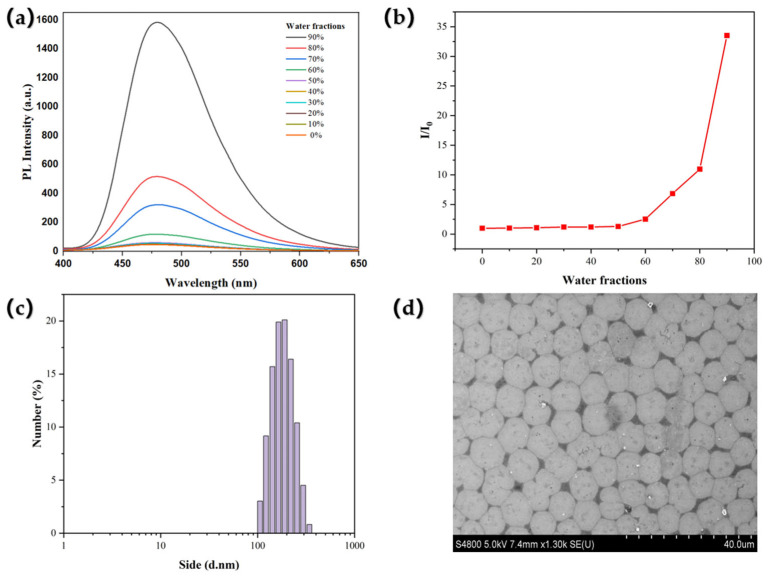
(**a**) Emission spectra of 2,5-2TPE-thiophene in THF/H_2_O mixtures (concentration = 10 μM. λ_ex_ = 365 nm). (**b**) Plots of the emission intensities of 2,5-2TPE-thiophene in THF/H_2_O mixtures versus water fractions (f_w_ = 0) at 480 nm. (**c**) The DLS plot of 2,5-2TPE-thiophene in 90% (vol%) H_2_O/THF solution. (**d**) SEM of 2,5-2TPE-thiophene in 90% (vol%) H_2_O/THF solution.

**Figure 4:**
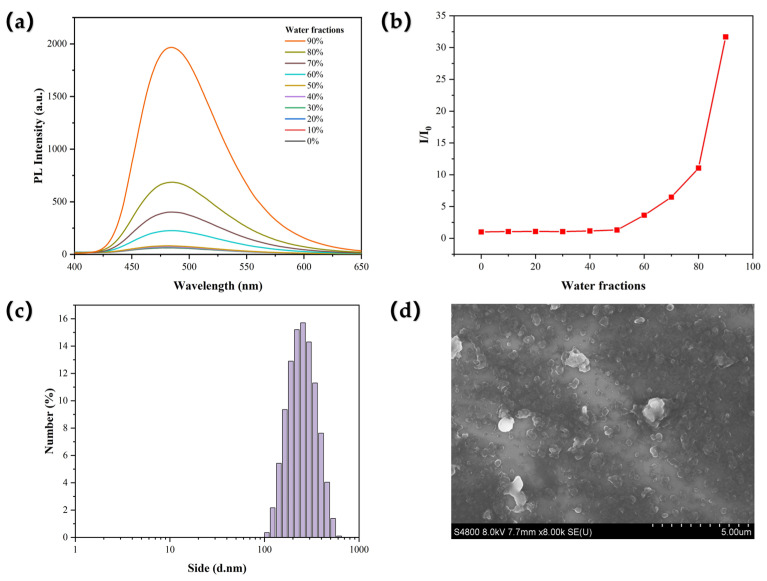
(**a**) Emission spectra of 2,3,5-3TPE-thiophene in THF/H_2_O mixtures (concentration = 10 μM. λ_ex_ = 338 nm). (**b**) Plots of the emission intensities of 2,3,5-3TPE-thiophene in THF/H_2_O mixtures versus water fractions (f_w_ = 0) at 484 nm. (**c**) The DLS plot of 2,3,5-3TPE-thiophene in 90% (vol%) H_2_O/THF solution. (**d**) SEM of 2,3,5-3TPE-thiophene in 90% (vol%) H_2_O/THF solution.

All four compounds exhibited typical AIE characteristics, wherein fluorescence intensity increased with increasing water fraction (Figure 1, Figure 2, Figure 3 and Figure 4). This is attributed to the increased presence of poor solvents (water), promoting molecular aggregation and subsequently restricting intramolecular rotations, a mechanism characteristic of the AIE effect.

To compare their fluorescent properties, absolute photoluminescence quantum yields (PLQYs) were measured at their solid state, as summarized in Table 1. Interestingly, among the four regioisomers, the 2-TPE-thiophene shows the highest PLQY at 52.86%. In contrast, both 2,5-2TPE-thiophene and 2,3,5-3TPE-thiophene, which contain two and three TPE substituents, respectively, displayed significantly lower PLQYs of approximately 22%. The 3-TPE-thiophene has the lowest PLQY, measured at only 13.07%. The result does not fit with our previous prediction that increasing the TPE part would enhance fluorescence efficiency, suggesting that additional structural factors may govern photophysical behavior. To elucidate the underlying reasons for these unexpected trends, a detailed single-crystal X-ray diffraction analysis was conducted, as shown in Section 2.4.

### 2.3. Thermal Properties

To investigate the thermal stability of four compounds, a thermogravimetric analysis (TGA) was performed (Figures S13–S16). The data are summarized in Table 1. The thermal decomposition temperature (Td, corresponding to 5% weight loss) of 2-TPE-thiophene and 3-TPE-thiophene is similar, around 207 °C. The thermal decomposition temperature (Td, corresponding to 5% weight loss) of 2,5-2TPE-thiophene and 2,3,5-3TPE-thiophene is much more thermally stable than that of monosubstituted ones, the latter being above 400 °C. The enhanced stability is attributed to the extended π electron delocalization. The result demonstrates that 2,5-2TPE-thiophene and 2,3,5-3TPE-thiophene both have good thermal stability, promoting their potential in optical materials.

### 2.4. Single-Crystal Structure Analysis

**Figure 5:**
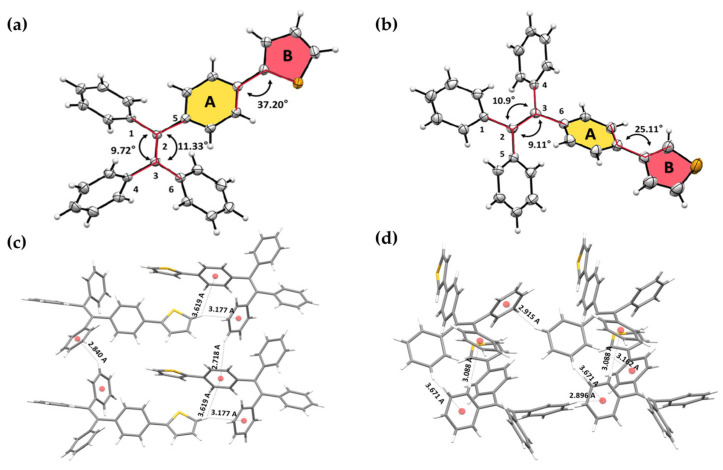
(**a**,**b**) View of the Oak Ridge Thermal Ellipsoid plot of the single-crystal molecular structures of 2-TPE-thiophene and 3-TPE-thiophene. (**c**,**d**): Packing mode and multiple C-H···π interactions in a single crystal cell of 2-TPE-thiophene and 3-TPE-thiophene (sulfur atoms are marked with yellow color).

**Figure 6:**
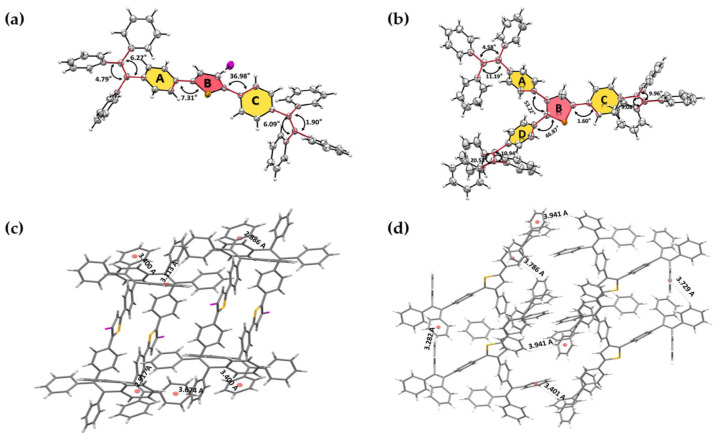
(**a**,**b**) View of the Oak Ridge Thermal Ellipsoid plot of the single-crystal molecular structures of 2,5-2TPE-thiophene and 2,3,5-3TPE-thiophene. (**c**,**d**): Packing mode and multiple C-H···π interactions in a single crystal cell of 2,5-2TPE-thiophene and 2,3,5-3TPE-thiophene (sulfur atoms are marked with yellow color, and Bromo atoms are marked with purple color).

To better understand their difference in fluorescent properties, the single-crystal structures are needed. By diffusing methanol into dichloromethane or chloroform solution, single crystals of 2-TPE-thiophene, 3-TPE-thiophene, and 2,3,5-3TPE-thiophene can be obtained. The single crystal of 2,5-2TPE-thiophene was obtained from ethyl acetate solution evaporation. The crystal structures of 2-TPE-thiophene, 3-TPE-thiophene, 2,5-2TPE-thiophene, and 2,3,5-3TPE-thiophene are shown in Figure 5 and Figure 6, and the detailed crystal data are listed in Table S1.

As shown in Figure 5a,b the single-crystal structures of the monosubstituted TPE–thiophene derivatives exhibit notable differences, particularly in the dihedral angles. Specifically, the dihedral angles of the central carbon–carbon double bond within the TPE core, as well as those between the thiophene ring and its adjacent phenyl ring, differ significantly between the two compounds. The 2-TPE-thiophene molecule displays larger dihedral angles at the TPE core and more pronounced twisting between the thiophene and neighboring phenyl ring compared to 3-TPE-thiophene. These structural distinctions lead to divergent molecular packing arrangements and variations in non-covalent interactions, particularly C–H···π interactions, as illustrated in Figure 5c,d.

As shown in Figure 6, the dihedral angles of the central carbon–carbon double bonds within the TPE cores, as well as those between the thiophene ring and adjacent phenyl rings, were analyzed for 2,5-2TPE-thiophene and 2,3,5-3TPE-thiophene. Owing to the presence of multiple TPE substituents, these multi-substituted derivatives exhibit more complex conformational geometries compared to their monosubstituted counterparts. Nevertheless, the overall molecular distortion in both 2,5-2TPE-thiophene and 2,3,5-3TPE-thiophene appears to be less pronounced than that observed in 2-TPE-thiophene. A more detailed structural and photophysical correlation analysis is presented in the following section.

## 3. Discussion

**Figure 7:**
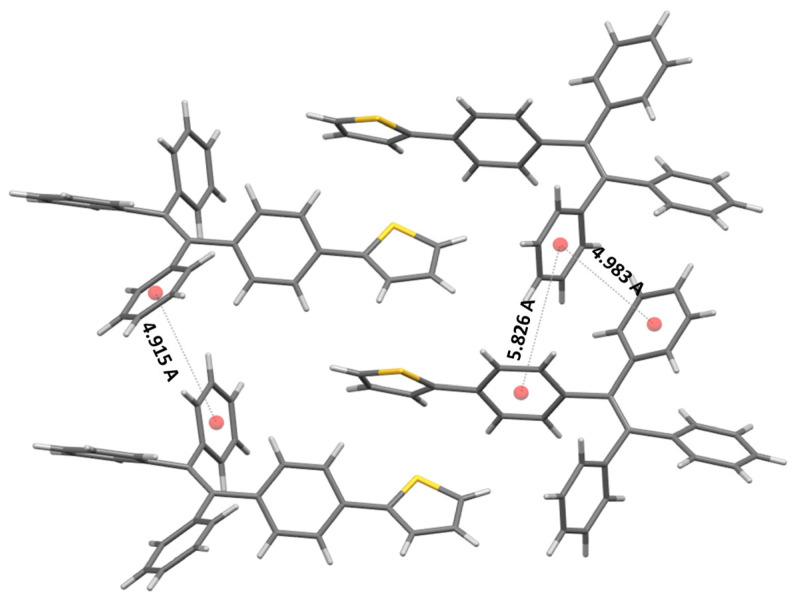
Packing mode of compound 2-TPE-thiophene.

**Figure 8:**
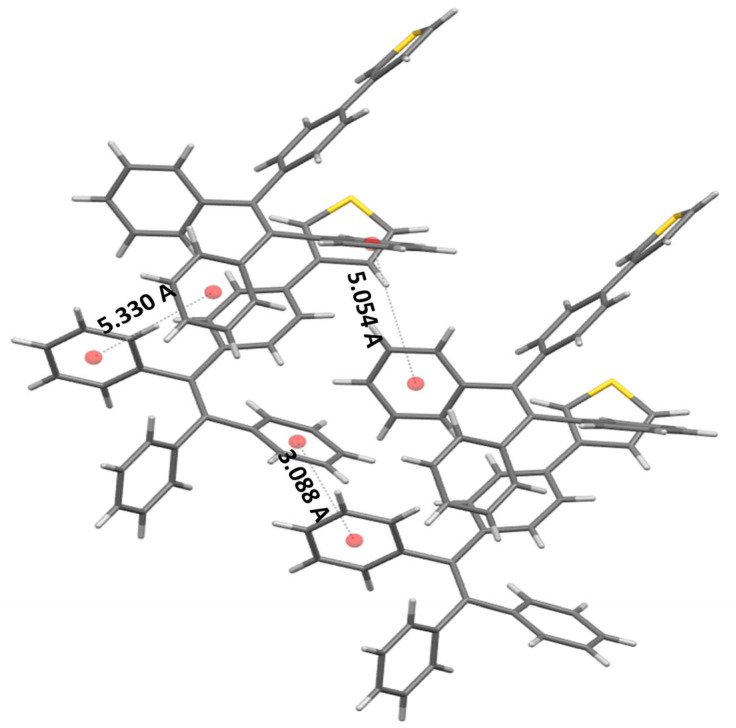
Packing mode of compound 3-TPE-thiophene.

Previous studies have established that intramolecular interactions among the four phenyl rings of the TPE subunit are critical determinants of photoluminescence performance [26]. A comparative analysis of 2-TPE-thiophene and 3-TPE-thiophene (Figure 5, Figure 7 and Figure 8) reveals distinct differences in their intramolecular interactions. Specifically, 2-TPE-thiophene exhibits stronger C–H···π interactions and weaker π–π stacking compared to 3-TPE-thiophene. The enhanced C–H···π interactions in 2-TPE-thiophene contribute to a greater torsional distortion of the TPE core, reducing molecular planarity and thereby suppressing π–π stacking. This structural feature is closely associated with its high PLQY, as excessive π–π stacking is known to quench emission in solid states [27,28,29,30].

**Figure 9:**
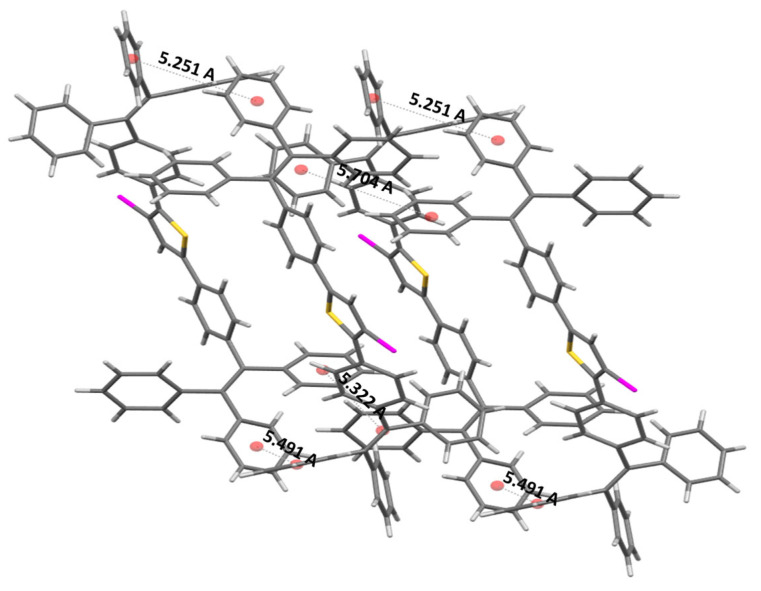
Packing mode of compound 2,5-2TPE-thiophene.

**Figure 10:**
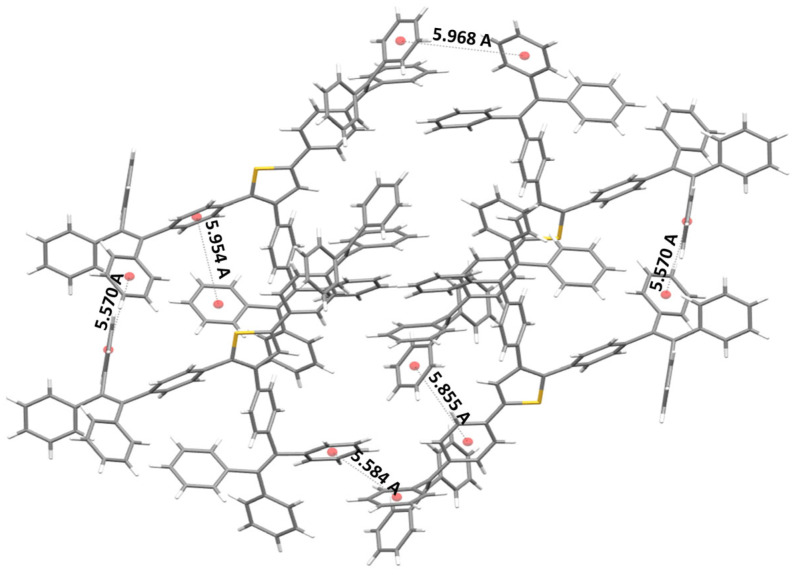
Packing mode of compound 2,3,5-3TPE-thiophene.

Contrary to initial expectations, increasing the number of TPE substituents in the thiophene core did not enhance the PLQY; rather, it led to reduced emission efficiency. Both 2,5-2TPE-thiophene (Figure 9) and 2,3,5-3TPE-thiophene (Figure 10) show similar levels of C–H···π and π–π interactions, which are weaker and stronger, respectively, than those observed in 2-TPE-thiophene. This similarity in intermolecular interaction profiles explains their comparable PLQYs, which are notably lower than that of the monosubstituted 2-TPE-thiophene.

To further verify our analysis, theoretical calculations were performed using density functional theory (DFT) at the B3LYP/6-31G(d) level in the gas phase. The optimized structure and the orbital distributions of the highest occupied molecular orbital (HOMO) and the lowest unoccupied molecular orbital (LUMO) energy levels are shown in Figures S17–S19. The orbitals from the thiophene part and the TPE parts are mainly attributed to both energy levels of all four compounds while the compound 2,3,5-3TPE-thiophene is similar to 2,5-2TPE-thiophene with only two TPE parts mainly attributing to the energy levels. That may be the reason why 2,5-2TPE-thiophene and 2,3,5-3TPE-thiophene show similar levels of C–H···π and π–π interactions in crystal packing mode, which is possibly caused by the steric hindrance effect between TPE and thiophene parts, leading to a similar PLQY in solid state. Meanwhile, the explanation for the PLQY difference between 2-TPE-thiophene and 3-TPE-thiophene is mainly due to the electronic effect, similarly so for the compounds discussed in our previous paper [26].

These findings underscore that the primary factor influencing the PLQY in TPE–thiophene derivatives is not the number of TPE units, but rather the nature and extent of intramolecular interactions—particularly those governed by the dihedral angles between the central carbon–carbon double bond of the TPE core and the adjacent thiophene–phenyl junctions. Therefore, the rational control of molecular conformation, rather than simply increasing AIE-active units, is essential for the design of highly luminescent TPE-based materials.

## 4. Materials and Methods

### 4.1. Materials

Methanone, (4-bromophenyl)phenyl-(AR) was acquired from Adamas; Tetrakis(triphenylphosphine)palladium (AR) was acquired from Adamas; Benzophenone (AR) was acquired from Adamas; Sodium Sulfate Anhydrous (AR) was acquired from Aladdin; Thiophene-2-boronic acid (AR) was acquired from Adamas; Thiophene-3-boronic acid (AR) was acquired from Adamas; Tetrahydrofuran (AR) was acquired from Adamas; Dichloromethane (AR) was acquired from Adamas; Ethyl acetate (AR) was acquired from Adamas; Petroleumether (AR) was acquired from Adamas; 1,4-Dioxane (AR) was acquired from Adamas; N-hexane (AR) was acquired from Adamas; Carbonic acid, dipotassium salt (AR) were acquired from General-reagent; 2,3,5-Tribromothiophene (AR) was acquired from Adamas; Bis(pinacolato)diborane (AR) was acquired from Adamas; Potassium Acetate (AR) was acquired from Adamas.

### 4.2. Methods

#### 4.2.1. Synthesis of 2-TPE-Thiophene

A mixture of 1-(4-bromophenyl)triphenylethene (500 mg, 1.22 mmol), thiophene-2-boronic acid (233.29 mg, 1.82 mmol), K_2_CO_3_ (671.97 mg, 4.86 mmol), and Pd(PPh_3_)_4_ (70.23 mg, 0.06 mmol) was dissolved in distilled water (4 mL) and THF (12 mL) under nitrogen. The resulting mixture was stirred at 80 °C for 24 h. After cooling to room temperature, the mixture was extracted with dichloromethane, dried with anhydrous Na_2_SO_4_, and filtered. The filtrates were concentrated under reduced pressure, and the crude product was purified by silica-gel column chromatography using a mixture of ethyl acetate/petroleum ether (1:4, *v/v*) as an eluent to give the compound 2-TPE-thiophene (360 mg, 71.6%) as a white solid. ^1^H NMR (400 MHz, CDCl_3_) δ(ppm): 7.37–7.35 (d, 2H), 7.22 (s, 1H), 7.12–7.02 (m, 19H). ^13^C NMR (400 MHz, CDCl_3_) δ(ppm): 132, 131.55, 131.48, 128.11, 127.96, 127.85, 127.78, 126.70, 126.66, 126.60, 125.15, 124.72, 123.01. HRMS: *m*/*z* calcd for [M + H]^+^ C30H22S1, 414.1442, found 414.1422.

#### 4.2.2. Synthesis of 3-TPE-Thiophene

A mixture of 1-(4-bromophenyl)triphenylethene (500 mg, 1.22 mmol), thiophene-3-boronic acid (233.29 mg, 1.82 mmol), K_2_CO_3_ (671.97 mg, 4.86 mmol), and Pd(PPh_3_)_4_ (70.23 mg, 0.06 mmol) was dissolved in distilled water (4 mL) and THF (12 mL) under nitrogen. The resulting mixture was stirred at 80 °C for 24 h. After cooling to room temperature, the mixture was extracted with dichloromethane, dried with anhydrous Na_2_SO_4_, and filtered. The filtrates were concentrated under reduced pressure, and the crude product was purified by silica-gel column chromatography using a mixture of ethyl acetate/petroleum ether (1:4, *v/v*) as an eluent to give the compound 3-TPE-thiophene (310 mg, 60%) as a white solid. ^1^H NMR (400 MHz, CDCl_3_) δ(ppm): 7.397 (s, 1H), 7.360–7.342 (m, 4H), 7.108–7.031 (m, 17H). ^13^C NMR (400 MHz, CDCl_3_) δ(ppm): 143.82, 142.76, 142.09, 141.28, 140.65, 133.76, 131.96, 131.54, 131.48, 127.91, 127.82, 127.77, 126.62, 126.56, 126.24, 125.69, 120.15. HRMS: *m*/*z* calcd for [M + H]^+^ C30H22S1, 414.1442, found 414.1431.

#### 4.2.3. Synthesis of 2,5-2TPE-Thiophene

A mixture of 4,4,5,5-tetramethyl-2-(4-(1,2,2-triphenylvinyl)phenyl)-1,3,2 dioxaborolane (1.0 g, 2.18 mmol), 2,3,5-Tribromothiophene (175 mg, 0.54 mmol), K_2_CO_3_ (600 mg, 4.34 mmol), and Pd(PPh_3_)_4_ (100 mg, 0.086 mmol) was dissolved in distilled water (4.0 mL) and THF (20.0 mL) under nitrogen. The resulting mixture was stirred at 80 °C for 24 h. After cooling to room temperature, the mixture was extracted with dichloromethane, dried with anhydrous Na_2_SO_4_, and filtered. The filtrates were concentrated under reduced pressure, and the crude product was purified by silica-gel column chromatography using a mixture of hexane/DCM (3:1, *v/v*) as an eluent to give the compound 2,5-2TPE-thiophene (280 mg, 47.8%) as a white solid. ^1^ H NMR (400 MHz, CDCl_3_) δ(ppm): 7.33 (m, 2H), 7.30 (s, 1H), 7.22–7.20 (m, 3H), 7.13–7.02 (m, 33H). ^13^C NMR (400 MHz, CDCl_3_) δ(ppm): 132.11, 131.58, 131.53, 131.47, 127.96, 127.89, 127.79, 126.80, 126.69, 124.66. HRMS: *m*/*z* calcd for [M + H]^+^ C56H39S1Br1, 824.1935, found 824.19176.

#### 4.2.4. Synthesis of 2,3,5-3TPE-Thiophene

A mixture of 4,4,5,5-tetramethyl-2-(4-(1,2,2-triphenylvinyl)phenyl)-1,3,2 dioxaborolane (1.5 g, 3.27 mmol), 2,3,5-Tribromothiophene (100 mg, 0.32 mmol), K_2_CO_3_ (800 mg, 5.78 mmol), and Pd(PPh_3_)_4_ (150 mg, 0.14 mmol) was dissolved in distilled water (5.4 mL) and THF (27 mL) under nitrogen. The resulting mixture was stirred at 80 °C for 24 h. After cooling to room temperature, the mixture was extracted with dichloromethane, dried with anhydrous Na_2_SO_4_, and filtered. The filtrates were concentrated under reduced pressure, and the crude product was purified by silica-gel column chromatography using a mixture of hexane/DCM (3:1, *v/v*) as an eluent to give the compound 2,3,5-3TPE-thiophene (250 mg, 28.4%) as a white solid. ^1^H NMR (400 MHz, CDCl_3_) δ(ppm): 7.45–7.43 (m, 3H), 7.30–7.27 (m, 3H), 7.15–7.01 (m, 52H). ^13^C NMR (400 MHz, CDCl_3_) δ(ppm): 143.90–140.86, 139.01, 137.77, 132.38, 132.02, 131.55, 131.49, 128.42, 128.35, 127.95, 127.86, 127.82, 126.71, 126.66, 126.22, 124.72. HRMS: *m*/*z* calcd for [M + H]^+^ C82H58S1, 1075.4332, found 1075.42457.

#### 4.2.5. Computational Details

All geometries are optimized with the Gaussian 16 [31] software program at the B3LYP/6-31G(d) level in the gas phase. Molecular orbitals are generated and rendered with Gaussview 6.1 [32].

### 4.3. Characterization

^1^H NMR and ^13^C NMR spectra were obtained from a Bruker DRX400 (400 MHz) spectrometer (Rheinstetten, Germany). Chemical shifts were expressed in ppm (in chloroform-d (CDCl_3_)). TMS as an internal standard) and coupling constants (*J*) in Hz; UV-Vis Spectroscopy was performed on Thermo Scientific Genesys UV50 (Waltham, MA, USA) using standard quartz cuvettes (d = 1 cm); the mass spectroscopic was obtained by using a Bruker solanX 70 FT-MS (Rheinstetten, Germany); the fluorescence spectroscopy analysis of 2-TPE-thiophene and 3-TPE-thiophene was performed on Hitachi F-4600 FL Spectrophotometer (Tokyo, Japan). The fluorescence spectroscopy analysis of 2,5-2TPE-thiophene and 2,3,5-3TPE-thiophene was performed on Lengguang FL Spectrophotometer F97PRO (Shanghai, China); the absolute fluorescence quantum yields were tested using integrating sphere measurement by Hamamatsu spectrometer C11347-11 (Hamamatsu, Japan); particle size determination and disassembly tests were recorded using a Malvern ZETASIER Nano-ZS90 (Malvern, England), dynamic light scattering instrument at a fixed scattering angle of 90° and a temperature of 25 °C; TGA was conducted on a DTG-60H SHIMADZU (Kyoto, Japan) thermogravimetric analyzer heating from 30 °C to 800 °C at a ramp of 10 °C min^−1^ under a nitrogen flow; the FT-IR spectroscopy of the sample was investigated using a Thermo Scientific Nicolet iS10 FTIR Spectrometer (Waltham, MA, USA); the morphologies of the samples were investigated by scanning electron microscopy (SEM) using Hitachi S-4800 (Tokyo, Japan); single-crystal data of 2-TPE-thiophene and 3-TPE-thiophene were collected on a Bruker smart Apex (Rheinstetten, Germany) using a mirror-monochromated Cu Kα radiation (CCDC numbers: 2432370, 2432374). The single-crystal data of 2,5-2TPE-thiophene and 2,3,5-3TPE-thiophene were collected on a Bruker D8 venture diffractometer (Rheinstetten, Germany) with Ga radiation (CCDC numbers: 2434518, 2432395).

## 5. Conclusions

In this work, four regioisomeric TPE–thiophene derivatives were successfully synthesized by systematically varying the number and positions of TPE substituents on the thiophene core. Comprehensive photophysical investigations revealed that substitution patterns significantly influence the solid-state photoluminescence quantum yields (PLQYs). Notably, the ortho-monosubstituted compound (2-TPE-thiophene) exhibited the highest PLQY (52.86%), while increasing the number of TPE substituents, contrary to conventional expectations, did not enhance but instead reduced the PLQY.

Detailed single-crystal structure analyses demonstrated that the key factor governing emission efficiency lies in the molecular geometry, specifically, the dihedral angles of the carbon–carbon double bond located on the core of the TPE part. Larger dihedral distortions hinder π-π stacking and promote AIE activity, thus enhancing fluorescence. This study clearly establishes that the photophysical properties of TPE–thiophene systems are primarily dictated by regioisomeric configuration rather than merely the number of emissive units.

These findings offer new insights into the structure–property relationships of AIE-active materials and provide valuable design principles for the development of high-performance organic luminescent materials based on TPE and thiophene frameworks.

## Figures and Tables

**Table 1:** Photophysical properties of four TPE–thiophene derivatives.

Sample	λ_abs_ ^1^ (nm)	λ_em_ ^1^ (nm)	ε ^2^ (L/mol·cm)	Φ_F_ ^3^ (%)	Td ^4^ (^o^C)
2-TPE-thiophene	331	480	27,510	52.86	207
3-TPE-thiophene	321	482	21,380	13.07	208
2,5-2TPE-thiophene	365	477	25,272	22.64	400
2,3,5-3TPE-thiophene	338	481	52,548	21.49	431

^1^ λ_abs_ = the maximum absorption peak in THF solution, λ_em_ = the maximum emission peak in THF solution. ^2^ Molar extinction coefficients at λ_abs_ in THF. ^3^ Φ_F_ = Absolute PLQY in solid states. ^4^ Td = Temperature at 5% weight loss.

## Data Availability

The data that supports the results is confidential but can be made available upon reasonable request from other researchers.

## Supplementary Materials

The following supporting information can be downloaded at: https://www.mdpi.com/article/10.3390/molecules30142953/s1. Figures S1–S8 are the ^1^H and ^13^C NMR spectra of four compounds. Figures S9–S12 are the High-resolution mass spectra of four compounds. Figures S13–S16 are the thermalgravimetric analysis of four compounds. Figures S17–S19 are the orbit plots of the HOMO and LUMO of four compounds. Figures S20–S23 are the absorption spectra of four compounds in THF. Figures S24–S27 are the FT-IR spectra of four compounds. Table S1 is crystal data and structure refinements of four compounds.
