# Supplementary material for: Role of Substitution Patterns in Four Regioisomeric Tetraphenylethylene–Thiophene Derivatives
Source: Molecules. 2025 Jul 13;30(14):2953. doi: 10.3390/molecules30142953 (PMC12298548; doi:10.3390/molecules30142953)
Supplement: Supplementary file 1 [file molecules-30-02953-s001.zip › molecules-3702017-supplementary.pdf]

Supporting Information

Article

Role of Substitution Patterns in Four Regioisomeric Tetraphenylethylene–Thiophene Derivatives

**Shuai Hou [1], Hanxiao Tian [1], Ruiyao Li [1], Zishuai Huang [1], Dongyuan Zhu [1], Fan Xiao [1], Yunmeng Zhao [2,*]
and Jingjing Xu [1,*]**

[1] The Education Ministry Key Lab of Resource Chemistry, Shanghai Key Laboratory of Rare Earth Functional Materials, Shanghai Frontiers Science Center of Biomimetic Catalysis, College of Chemistry and Materials Science, Shanghai Normal University, Shanghai 200234, China; h1026712305@163.com (S.H.); thx2218@163.com (H.T.); 1000549716@smail.shnu.edu.cn (R.L.); 13782223290@163.com (Z.H.); 13619559574@163.com (D.Z.); 1000502140@smail.shnu.edu.cn (F.X.)

[2] Key Laboratory of Smart Manufacturing in Energy Chemical Process, Ministry of Education, East China University of Science and Technology, Shanghai 200237, China

* Correspondence: yunmeng.zhao@ecust.edu.cn (Y.Z.); jingjingxu@shnu.edu.cn (J.X.)

CRediT authorship contribution statement

Conceptualization, J.X.; methodology, S.H. and H.T.; formal analysis, S.H., H.T., R.L. and J.X.; investigation, S.H., F.X. and D.Z.; data curation, Z.H.; writing—original draft preparation, S.H., Y.Z. and J.X.; writing—review and editing, Y.Z. and J.X.; visualization, S.H., H.T. and J.X.; supervision, J.X.; project administration, J.X.; funding acquisition, J.X. All authors have read and agreed to the published version of the manuscript.

Materials

Unless otherwise noted, all materials and reagents, including dry solvents, were obtained from commercial suppliers and used without further purification. All work-up processing and purification procedures were carried out using reagent-grade solvents in air.

Nuclear Magnetic Resonance

^{1}H NMR and ^{13}C NMR spectra were obtained from a Bruker DRX400 (400 MHz) spectrometer (Rheinstetten, Germany). Chemical shifts were expressed in ppm (in chloroform-d ($CDCl_3$)), TMS as an internal standard, and coupling constants (*J*) in Hz.

Mass Spectrometry

The mass spectroscopy was obtained by using a Bruker solanX 70 FT-MS (Rheinstetten, Germany).

UV-Vis Spectroscopy

UV-Vis Spectroscopy was performed on a Thermo Scientific Genesys UV50 (Waltham, MA, USA) using standard quartz cuvettes (d = 1 cm).

Fluorescence Spectroscopy

Fluorescence Spectroscopy of **2-TPE-thiophene** and **3-TPE-thiophene** were performed on an Hitachi F-4600 FL Spectrophotometer (Tokyo, Japan). Fluorescence Spectroscopy of **2,5-2TPE-thiophene** and **2,3,5-3TPE-thiophene** were performed on Shanghai Lengguang FL Spectrophotometer F97PRO (Shanghai, China). A MacroFluorescence Cuvette was used with a Stopper (diameter = 1 cm).

Absolute Fluorescence Quantum Yields

The absolute fluorescence quantum yields were tested using an integrating sphere measurement by Hamamatsu spectrometer C11347-11 (Hamamatsu, Japan).

Dynamic Light Scattering

Particle size determination and disassembly tests were recorded using a Malvern ZETASIER Nano-ZS90 (Malvern, England), dynamic light scattering instrument at a fixed scattering angle of 90° and a temperature of 25 °C.

Thermogravimetric Analysis

TGA was conducted on a DTG-60H SHIMADZU thermogravimetric analyzer (Kyoto, Japan) heating from 30 ^{o}C to 800 ^{o}C at a ramp of 10 ^{o}C min^{-1} under a nitrogen flow.

FT-IR spectroscopy (KBr disc)

The FT-IR spectroscopy of sample were investigated using Thermo Scientific Nicolet iS10 FTIR Spectrometer (Waltham, MA, USA).

Scanning Electron Microscopy

The morphologies of the samples were investigated by scanning electron microscopy (SEM) using a Hitachi S-4800 (Tokyo, Japan).

Crystallography

Single crystal data of **2-TPE-thiophene** and **3-TPE-thiophene** were collected on a Bruker smart Apex (Rheinstetten, Germany) using a mirror-monochromated Cu Kα radiation. Single crystal data of **2,5-2TPE-thiophene** and **2,3,5-3TPE-thiophene** were collected on a Bruker D8 venture diffractometer (Rheinstetten, Germany) with Ga radiation. The crystal was kept at 170.00 K during data collection.

Computational details

All geometries are optimized with Gaussian 16[1] software at B3LYP/6-31G(d) level in gas phase. Molecular orbitals are generated and rendered with Gaussview 6.1[2].

1 Synthesis

1.1 Synthesis of 2-TPE-thiophene

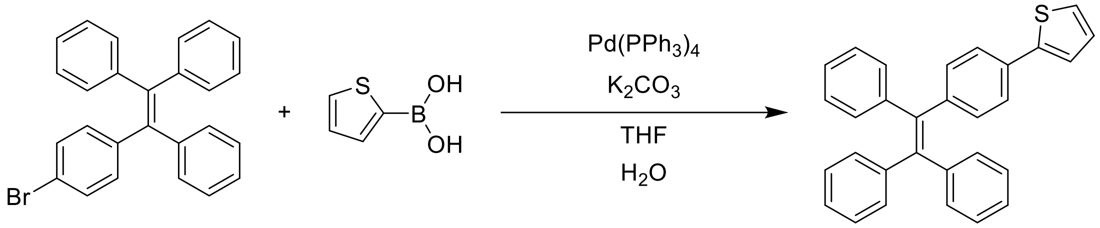

1-(4-bromophenyl)triphenylethene was synthesized according to the literature[3].

A mixture of 1-(4-bromophenyl)triphenylethene (500 mg, 1.22 mmol), thiophene-2-boronic acid (233.29 mg, 1.82 mmol), K_2CO_3 (671.97 mg, 4.86 mmol) and $Pd(PPh_3)_4$ (70.23 mg, 0.06 mmol) were dissolved in distilled water (4 mL) and THF (12 mL) under nitrogen. The resulting mixture was stirred at 80 °C for 24 h. After cooling to room temperature, the mixture was extracted with dichloromethane, dried with anhydrous Na_2SO_4, and filtered. The filtrates were concentrated under reduced pressure and the crude product was purified by silica-gel column chromatograph using a mixture of ethyl acetate /petroleum ether (1: 4, v/ v) as eluent to give compound 2-TPE-thiophene (360 mg, 71.6%) as white solid. ^1H NMR (400 MHz, $CDCl_3$) δ(ppm): 7.37–7.35 (d, 2H), 7.22 (s, 1H), 7.12–7.02 (m, 19H). ^{13}C NMR (400 MHz, $CDCl_3$) δ(ppm): 132, 131.55, 131.48, 128.11, 127.96, 127.85, 127.78, 126.70, 126.66, 126.60, 125.15, 124.72, 123.01. HRMS: m/z calcd for [M+H]+ C30H22S1, 414.1442, found 414.1422.

1.2 Synthesis of 3-TPE-thiophene

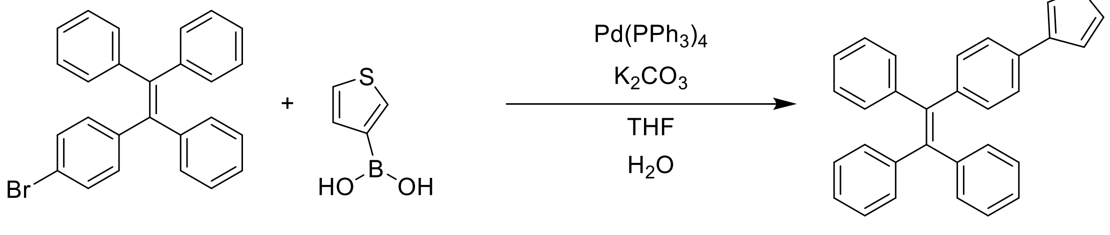

A mixture of 1-(4-bromophenyl)triphenylethene (500 mg, 1.22 mmol), thiophene-3-boronic acid (233.29

mg, 1.82 mmol), K_2CO_3 (671.97 mg, 4.86 mmol) and $Pd(PPh_3)_4$ (70.23 mg, 0.06 mmol) were dissolved in distilled water (4 mL) and THF (12 mL) under nitrogen. The resulting mixture was stirred at 80 °C for 24 h. After cooling to room temperature, the mixture was extracted with dichloromethane, dried with anhydrous Na_2SO_4, and filtered. The filtrates were concentrated under reduced pressure and the crude product was purified by silica-gel column chromatograph using a mixture of ethyl acetate /petroleum ether (1: 4, v/ v) as eluent to give compound 3-TPE-thiophene (310 mg, 60%) as white solid. ^1H NMR (400 MHz, $CDCl_3$) δ(ppm): 7.397 (s, 1H), 7.360–7.342 (m, 4H), 7.108–7.031 (m, 17H). ^{13}C NMR (400 MHz, $CDCl_3$) δ(ppm): 143.82, 142.76, 142.09, 141.28, 140.65, 133.76, 131.96, 131.54, 131.48, 127.91, 127.82, 127.77, 126.62, 126.56, 126.24, 125.69, 120.15. HRMS: m/z calcd for [M+H]+ C30H22S1, 414.1442, found 414.1431.

1.3 Synthesis of 2,5-2TPE-thiophene

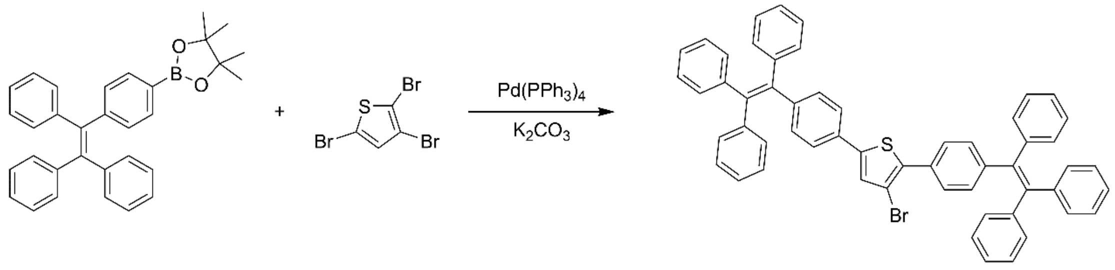

4,4,5,5-tetramethyl-2-(4-(1,2,2-triphenylvinyl)phenyl)-1,3,2 dioxaborolane was synthesised according to the literature[3].

A mixture of 4,4,5,5-tetramethyl-2-(4-(1,2,2-triphenylvinyl)phenyl)-1,3,2 dioxaborolane (1.0 g, 2.18 mmol), 2,3,5-Tribromothiophene (175 mg, 0.54 mmol), K_2CO_3 (600 mg, 4.34 mmol) and $Pd(PPh_3)_4$ (100 mg, 0.086 mmol) were dissolved in distilled water (4.0 mL) and THF (20.0 mL) under nitrogen. The resulting mixture was stirred at 80 °C for 24 h. After cooling to room temperature, the mixture was extracted with dichloromethane, dried with anhydrous Na_2SO_4, and filtered. The filtrates were concentrated under reduced pressure and the crude product was purified by silica-gel column

chromatograph using a mixture of hexane/DCM (3: 1, v/v) as eluent to give compound 2,5-2TPE-thiophene (280 mg, 47.8%) as white solid. ^{1}H NMR (400 MHz, $CDCl_3$) δ(ppm): 7.33 (m, 2H), 7.30 (s, 1H), 7.22–7.20 (m, 3H), 7.13–7.02 (m, 33H). ^{13}C NMR (400 MHz, $CDCl_3$) δ(ppm): 132.11, 131.58, 131.53, 131.47, 127.96, 127.89, 127.79, 126.80, 126.69, 124.66. HRMS: m/z calcd for [M+H]+ C56H39S1Br1, 824.1935, found 824.19176.

1.4 Synthesis of 2,3,5-3TPE-thiophene

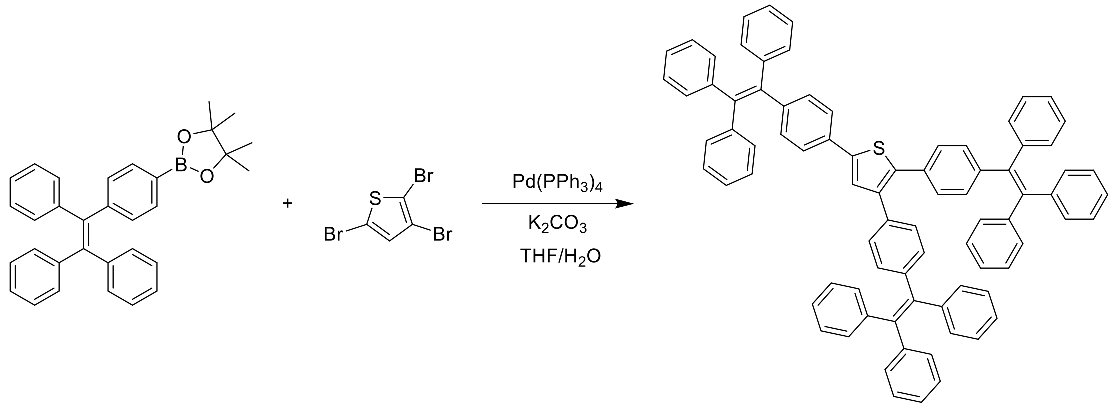

A mixture of 4,4,5,5-tetramethyl-2-(4-(1,2,2-triphenylvinyl)phenyl)-1,3,2 dioxaborolane (1.5 g, 3.27 mmol), 2,3,5-Tribromothiophene (100 mg, 0.32 mmol), K_2CO_3 (800 mg, 5.78 mmol) and $Pd(PPh_3)_4$ (150 mg, 0.14 mmol) were dissolved in distilled water (5.4 mL) and THF (27 mL) under nitrogen. The resulting mixture was stirred at 80 °C for 24 h. After cooling to room temperature, the mixture was extracted with dichloromethane, dried with anhydrous Na_2SO_4, and filtered. The filtrates were concentrated under reduced pressure and the crude product was purified by silica-gel column chromatograph using a mixture of hexane /DCM (3: 1, v/ v) as eluent to give compound 2,3,5-3TPE-thiophene (250 mg, 28.4%) as white solid. ^{1}H NMR (400 MHz, $CDCl_3$) δ(ppm): 7.45-7.43 (m, 3H), 7.30–7.27 (m, 3H), 7.15–7.01 (m, 52H). ^{13}C NMR (400 MHz, $CDCl_3$) δ(ppm): 143.90-140.86, 139.01,

137.77, 132.38, 132.02, 131.55, 131.49, 128.42, 128.35, 127.95, 127.86, 127.82, 126.71, 126.66, 126.22, 124.72.

HRMS: m/z calcd for [M+H]+ $C_{82}H_{58}S_1$, 1075.4332, found 1075.42457.

2 Analytical data

2.1 Nuclear Magnetic Resonance

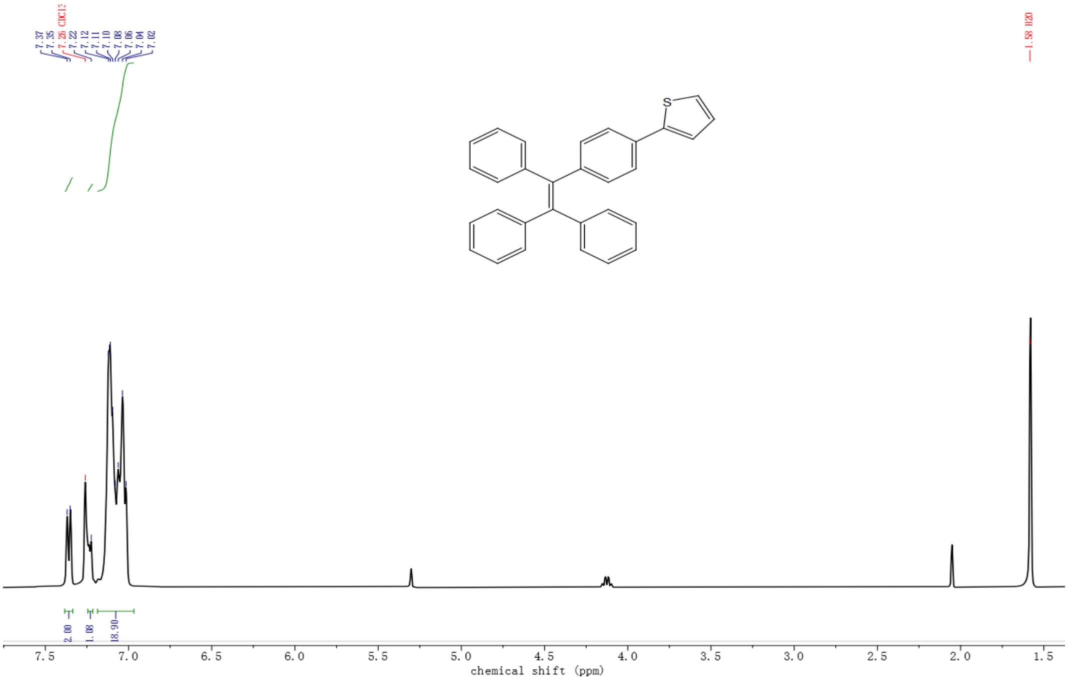

Figure S1. ^{1}H NMR spectrum of 2-TPE-thiophene (400 MHz, $CDCl_3$, 373.2 K).

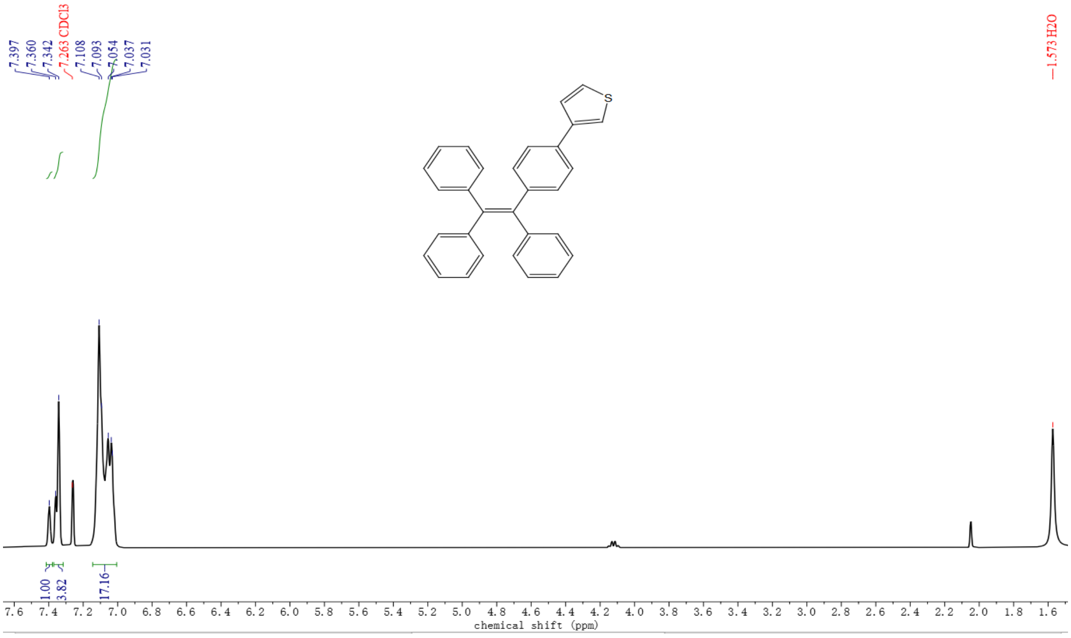

Figure S2. ^{1}H NMR spectrum of 3-TPE-thiophene (400 MHz, $CDCl_3$, 373.2 K).

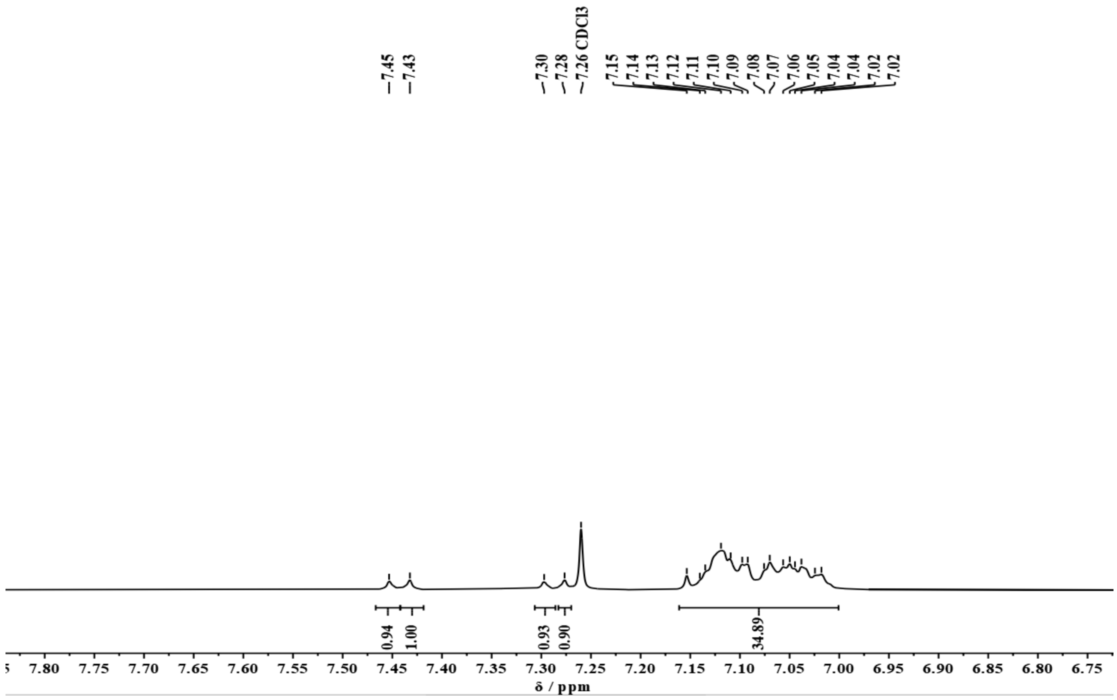

Figure S3. ^{1}H NMR spectrum of 2, 5-2TPE-thiophene (400 MHz, $CDCl_3$, 373.2 K).

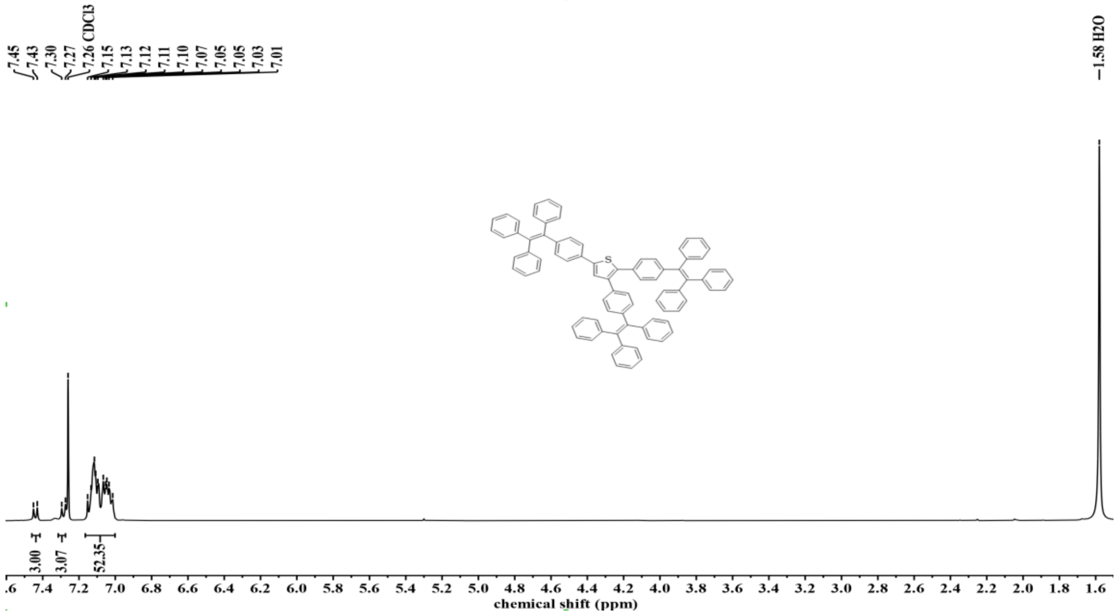

Figure S4. ^{1}H NMR spectrum of 2,3,5-3TPE-thiophene (400 MHz, $CDCl_3$, 373.2 K).

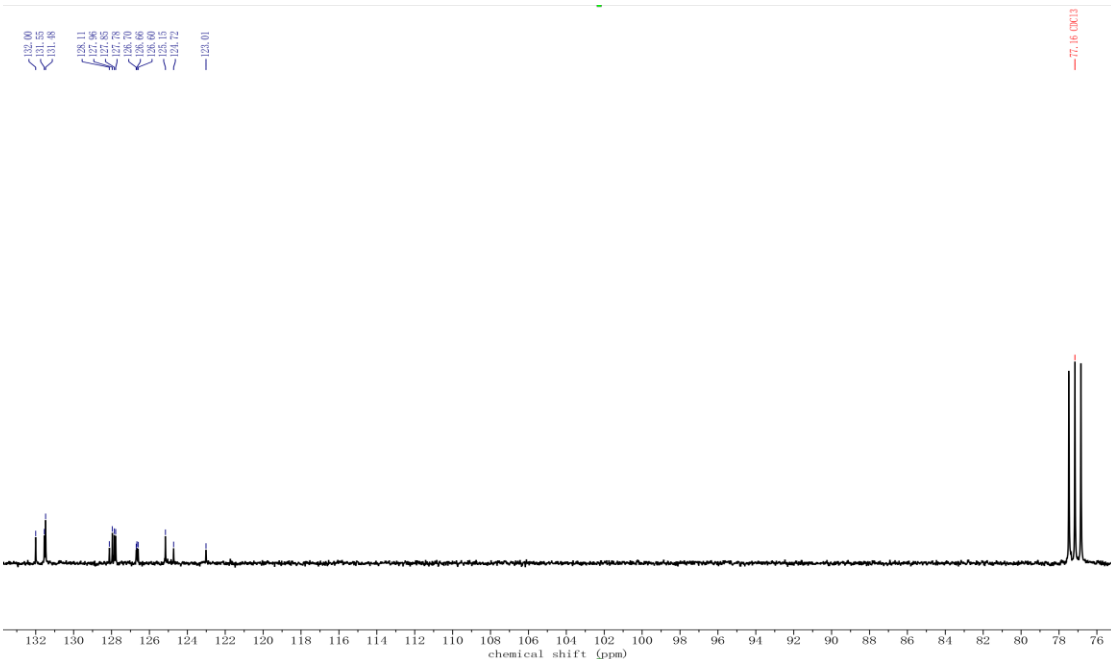

Figure S5. ^{13}C NMR spectrum of 2-TPE-thiophene (400 MHz, $CDCl_3$, 373.2 K).

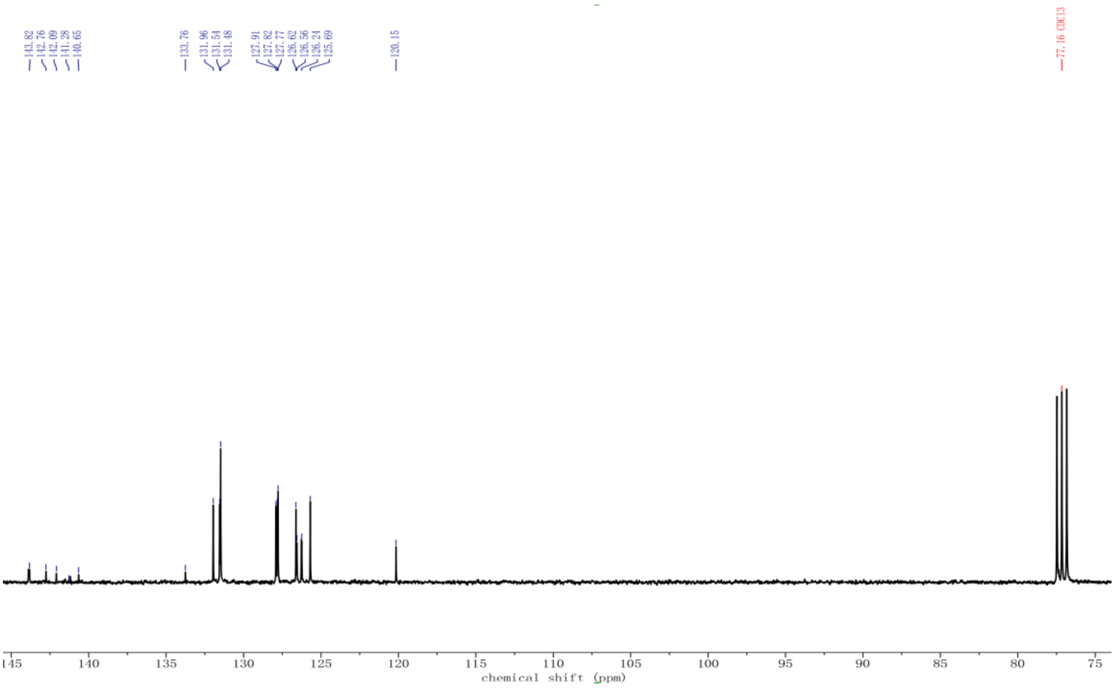

Figure S6. ^{13}C NMR spectrum of 3-TPE-thiophene (400 MHz, $CDCl_3$, 373.2 K).

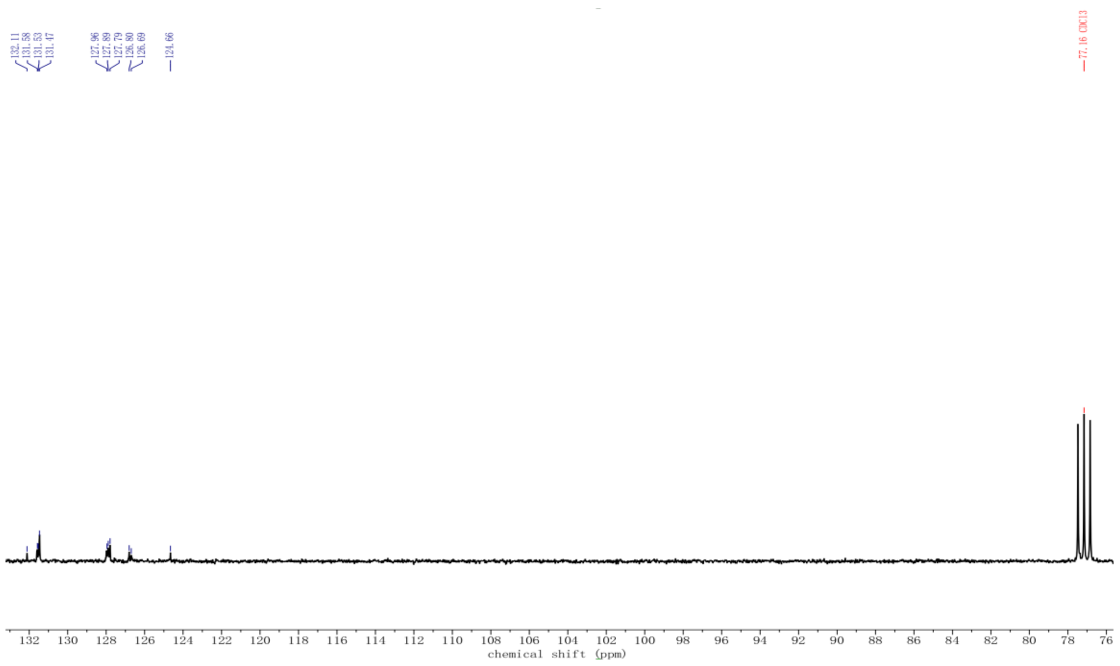

Figure S7. ^{13}C NMR spectrum of 2,5-2TPE-thiophene (400 MHz, $CDCl_3$, 373.2 K).

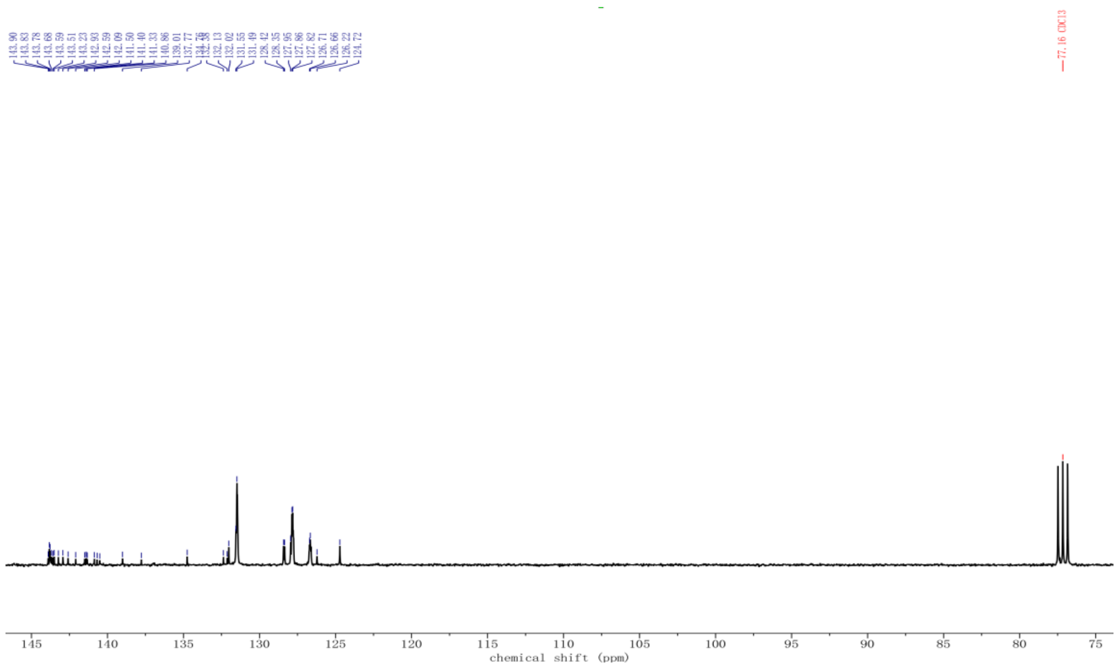

Figure S8. ^{13}C NMR spectrum of 2,3,5-3TPE-thiophene (400 MHz, $CDCl_3$, 373.2 K).

2.2 MS spectra

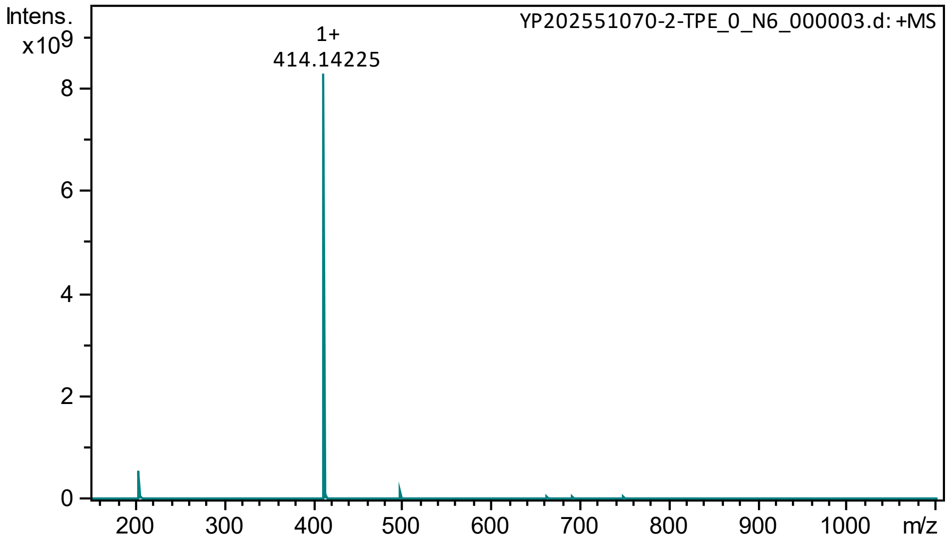

Figure S9. High-resolution mass spectra (HRMS) of compound 2-TPE-thiophene.

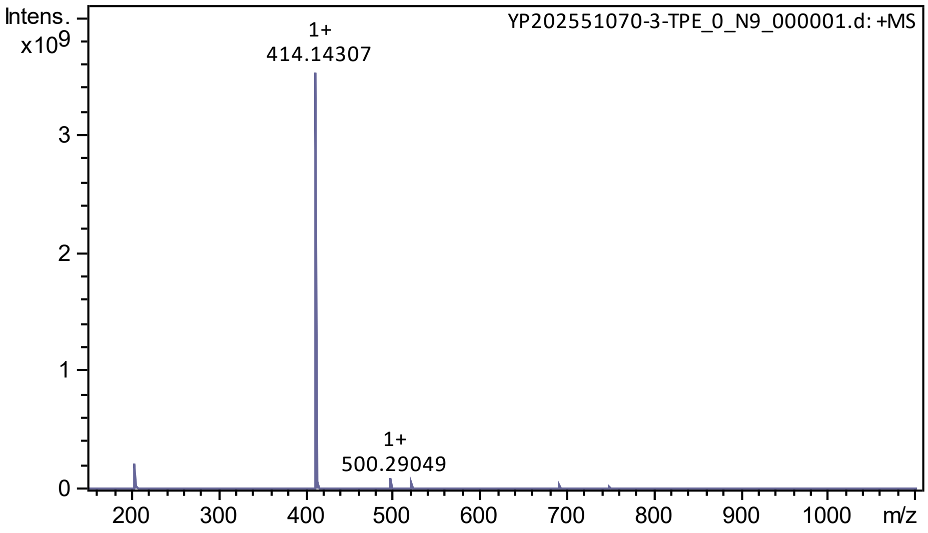

Figure S10. High-resolution mass spectra (HRMS) of compound 3-TPE-thiophene.

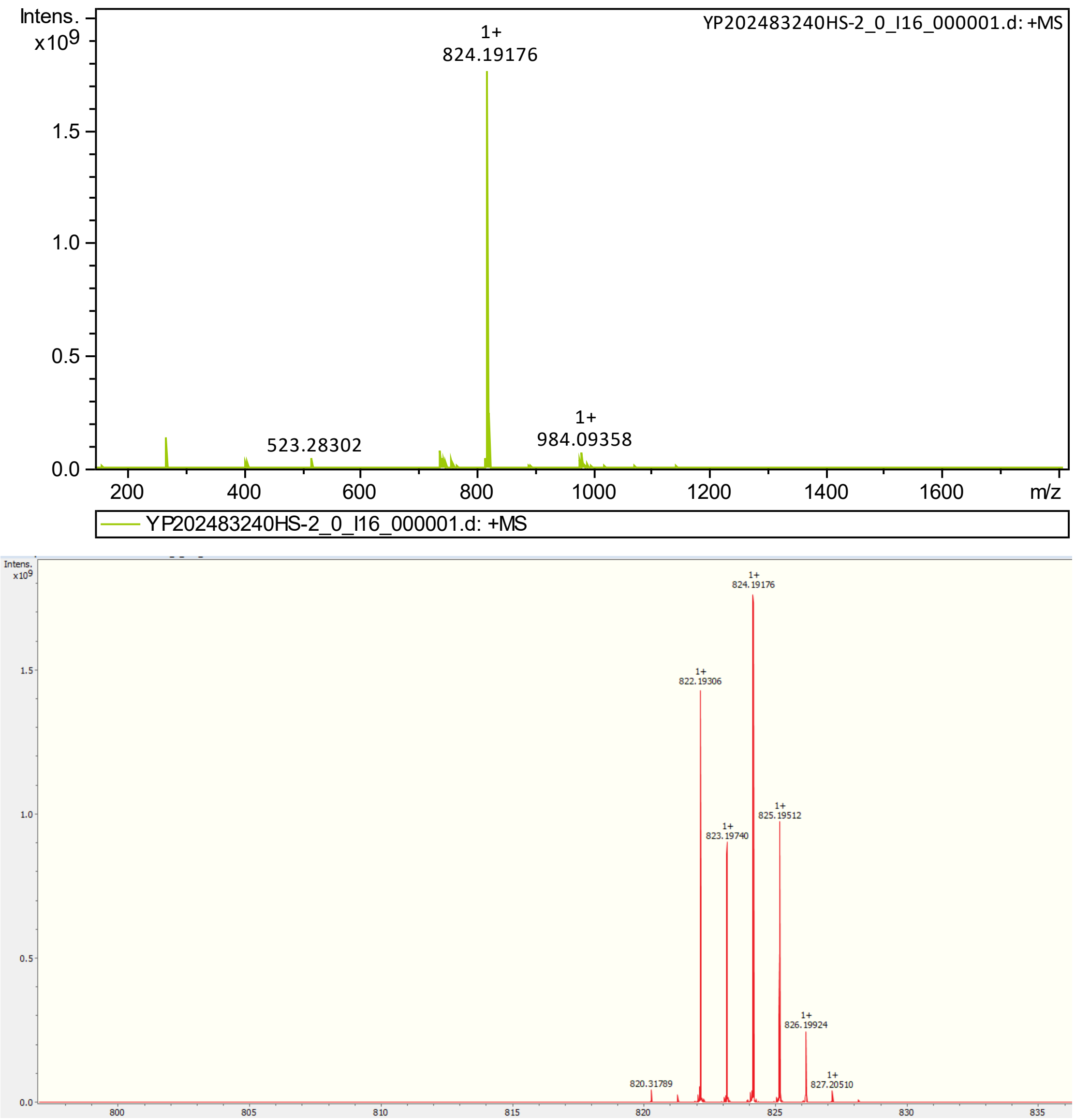

Figure S11. High-resolution mass spectra (HRMS) of compound 2,5-2TPE-thiophene.

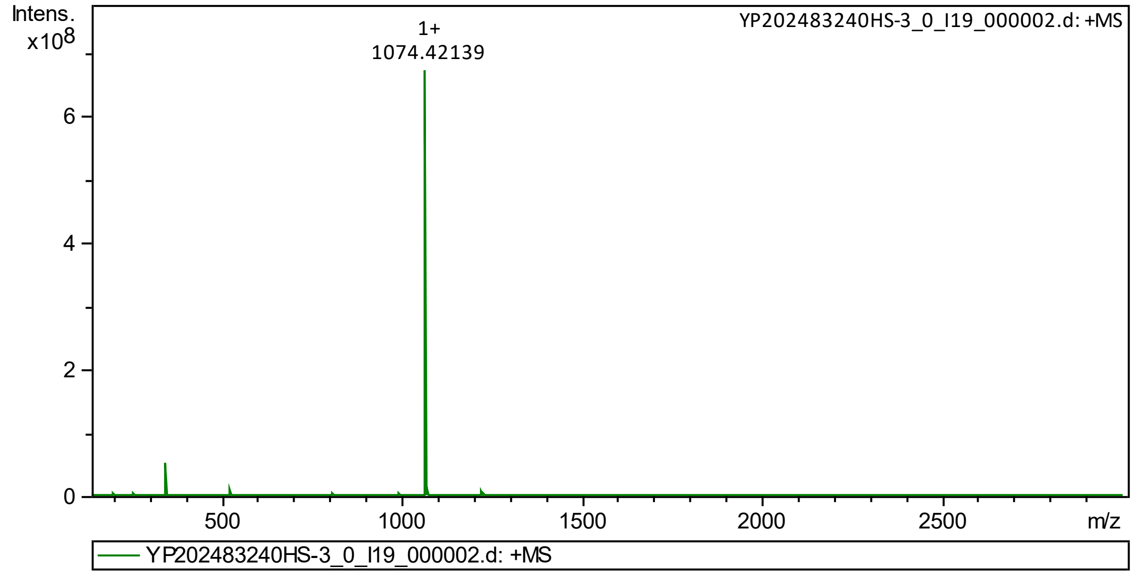

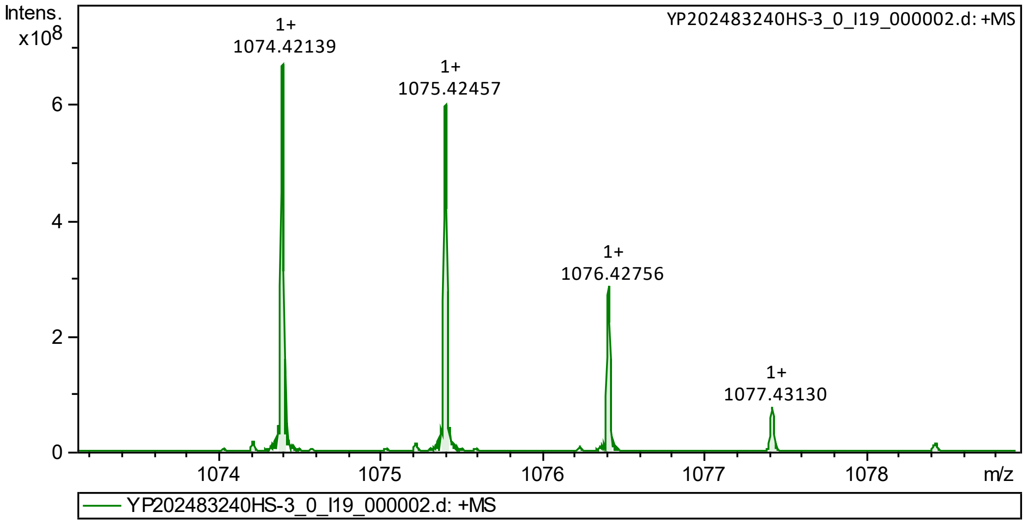

Figure S12. High-resolution mass spectra (HRMS) of compound 2,3,5-3TPE-thiophene.

2.3 Crystallography

Table. S1 Crystal data and structure refinements of four TPE-thiophene derivatives.

Compound	2-TPE-thiophene	3-TPE-thiophene	2 ,5-2TPE-thiophene	2,3,5-3TPE-thiophene
Empirical formula	$C_{30}H_{22}S_1$	$C_{30}H_{22}S_1$	$C_{56}H_{39}BrS_1$	$C_{82}H_{58}S_1$
Formula weight	414.53	414.53	881.92	1075.34
Temperature/K	173	173	173	170
Crystal system	monoclinic	monoclinic	triclinic'	monoclinic
Space group	P21/n	P 1 21 1	P -1	P 1 21/n 1
a/Å	9.4650(14)	9.9178(3)	9.432(3)	9.4556(2)
b/Å	9.8685(9)	9.2969(3)	11.230(4)	44.7255(9)
c/Å	47.078(8)	24.3927(6)	21.296(7)	14.1897(3)
α/°	90	90	84.450(13)	90

β/°	90.091(15)	99.214(1)	83.903(13)	90.776(1)
γ/°	90	90	88.295(12)	90
Volume/Å^3	4397.3(11)	2220.10(11)	2231.8(12)	6000.4(2)
ρ_{calc}g/cm^3	1.252	1.240	1.312	1.190
Z	8	4	2	4
μ/mm^{-1}	1.397	1.384	1.359	0.534
Goodness-of-fit on F2	1.053	1.038	1.017	1.016
Parameters	559	559	570	878
F(000)	1744.0	872.0	916.0	2264.0
Reflections collected	8038	8099	8441	7362
Final R indexes [I>=2σ (I)]	R1 = 0.0576, wR2 = 0.1782	R1 = 0.0525, wR2 = 0.1526	R1 = 0.0819, wR2 = 0.2538	R1 = 0.0581, wR2 = 0.1669

2.4 Thermogravimetric measurements

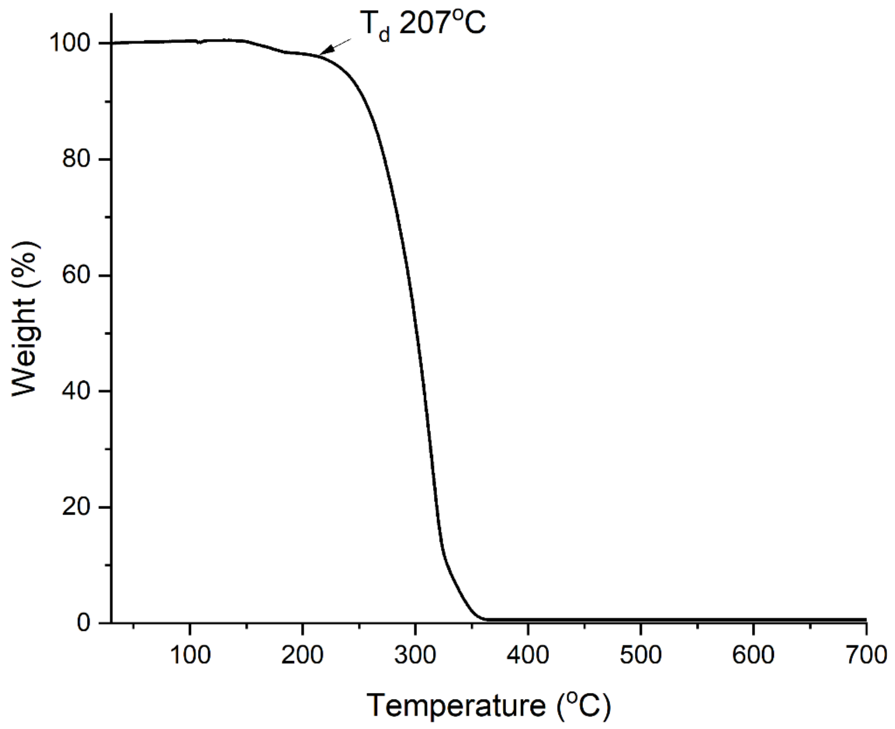

Figure S13. Thermalgravimetric analysis of 2-TPE-thiophene.

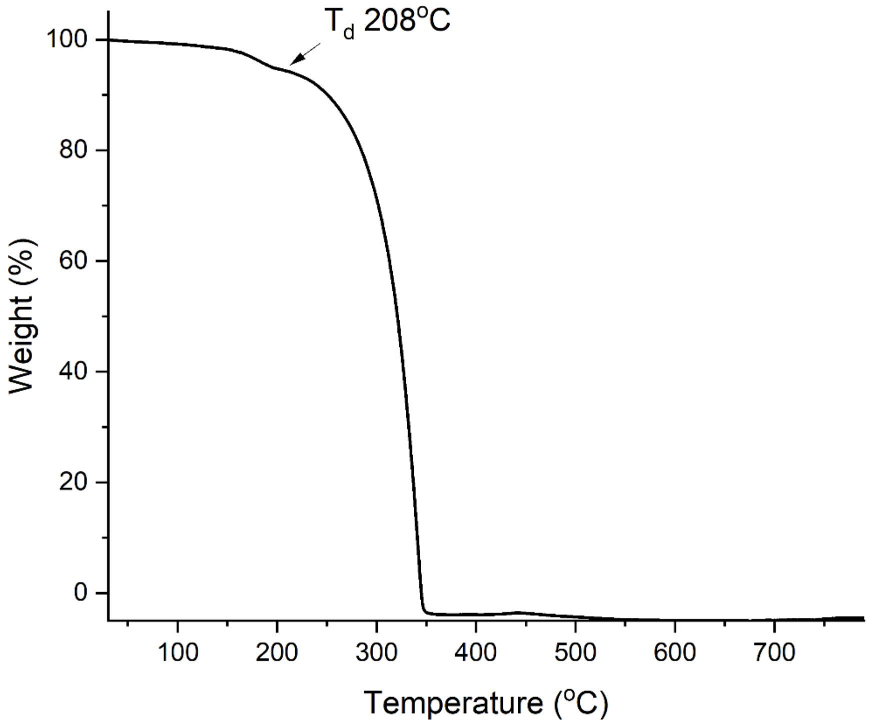

Figure S14. Thermalgravimetric analysis of 3-TPE-thiophene.

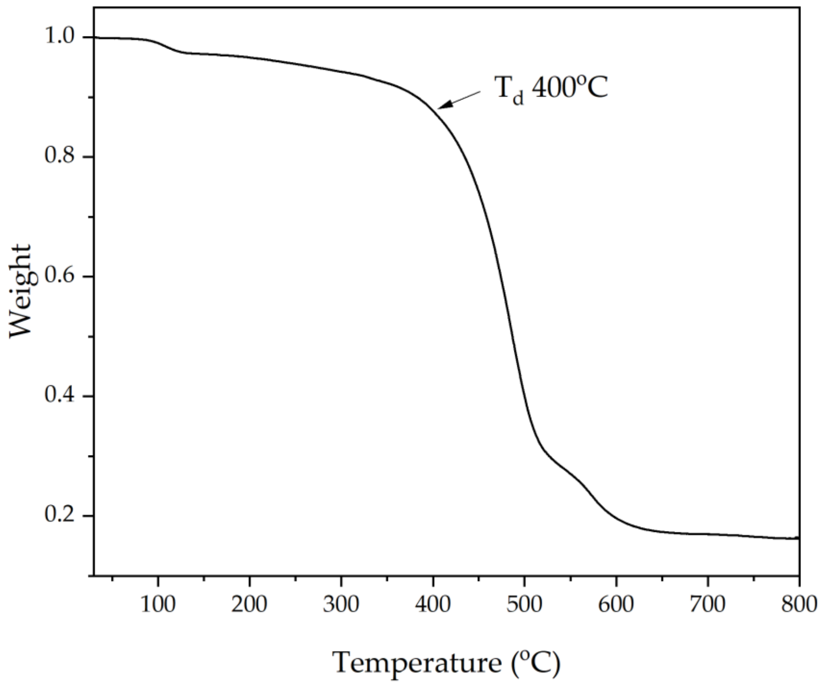

Figure S15. Thermalgravimetric analysis of 2, 5-2TPE-thiophene.

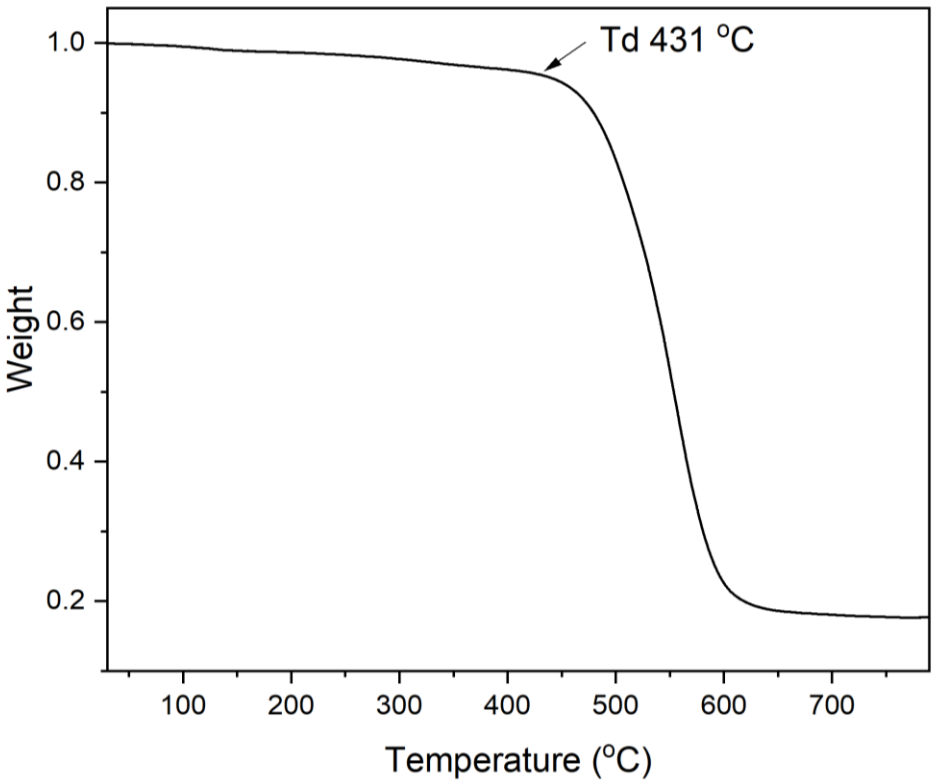

Figure S16. Thermalgravimetric analysis of 2,3,5-3TPE-thiophene.

2.5 DFT Calculations

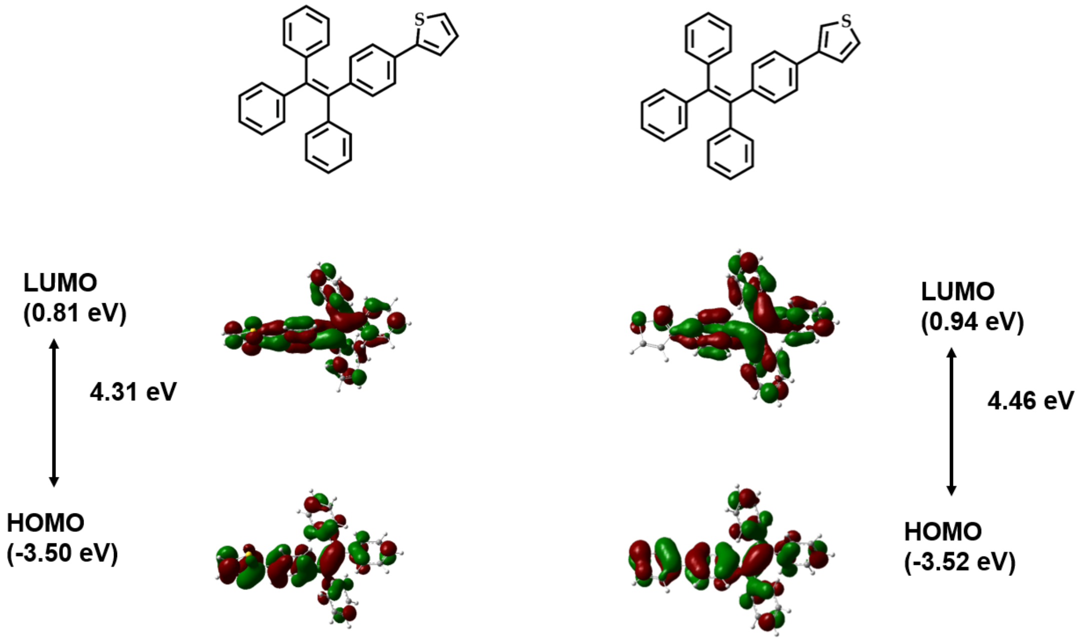

Figure S17. Orbit plot of the HOMO and LUMO of compound 2-thiophene-TPE and 3-thiophene-TPE.

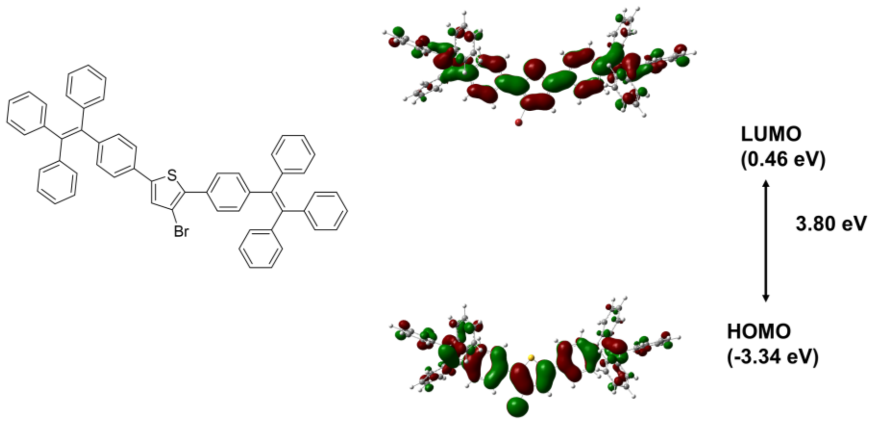

Figure S18. Orbit plot of the HOMO and LUMO of compound 2,5-2TPE-thiophene.

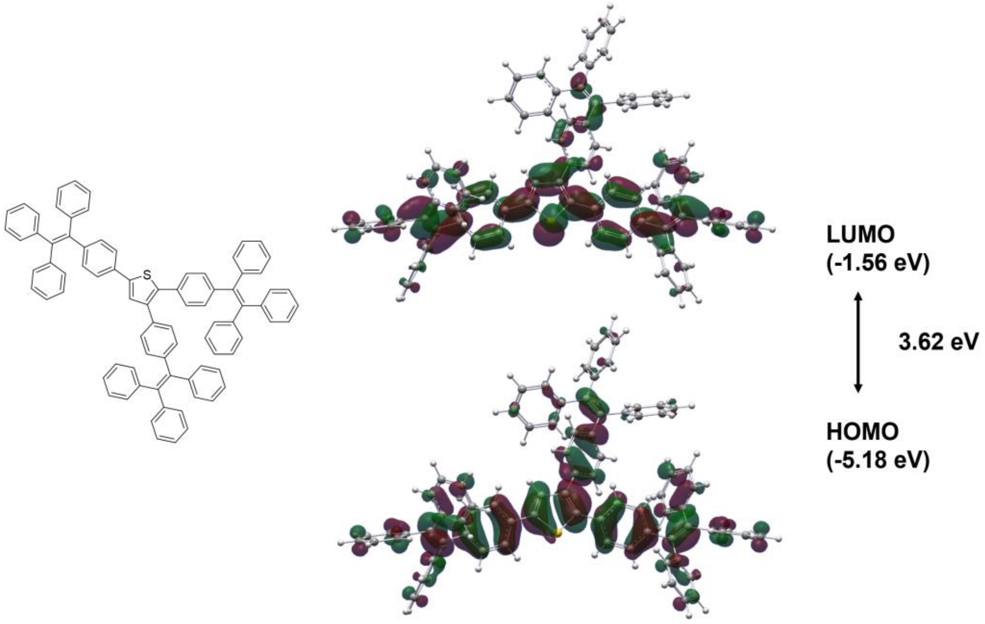

Figure S19. Orbit plot of the HOMO and LUMO of compound 2,3,5-3TPE-thiophene.

2.6 UV-Vis Spectroscopy

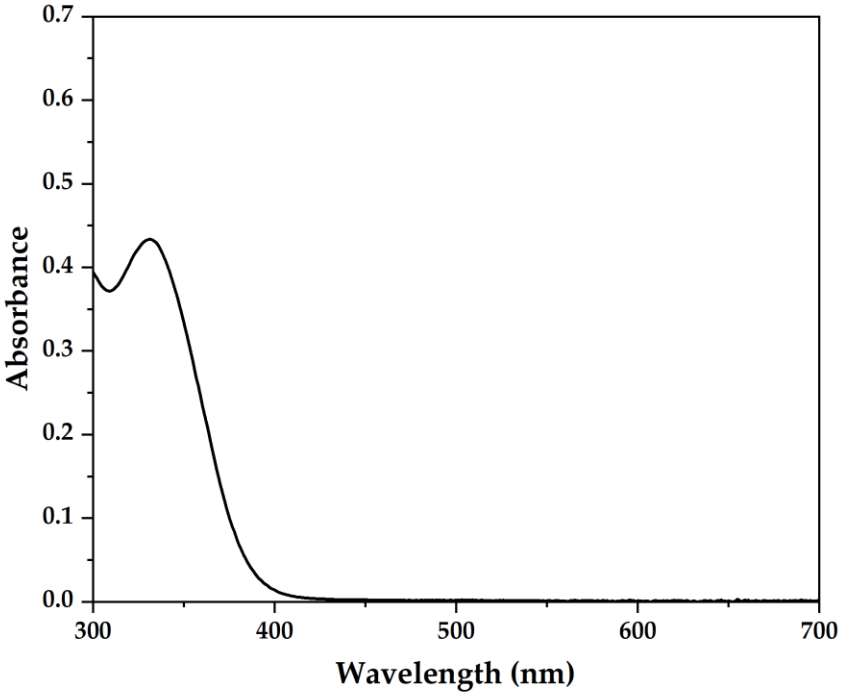

Figure S20. The absorption spectrum of 2-TPE-thiophene (10 uM) in THF.

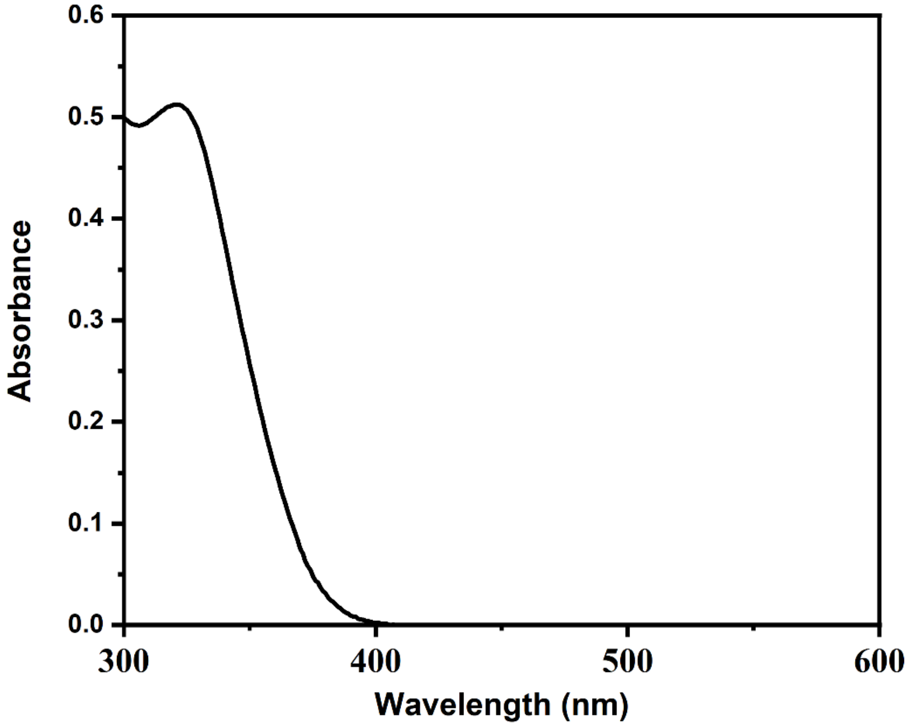

Figure S21. The absorption spectrum of 3-TPE-thiophene (10 uM) in THF.

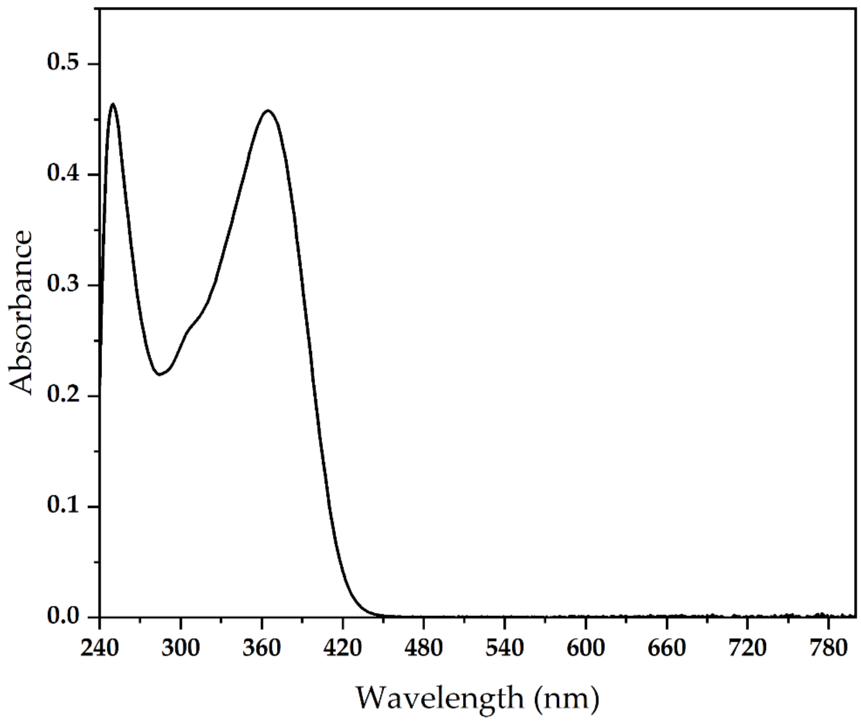

Figure S22. The absorption spectrum of 2,5-2TPE-thiophene (10 uM) in THF.

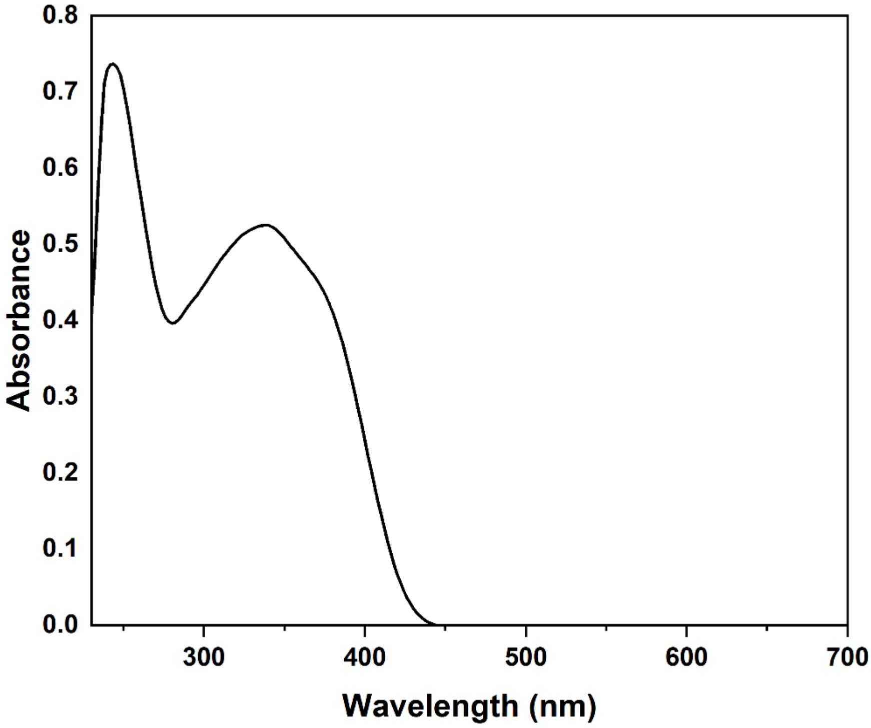

Figure S23. The absorption spectrum of 2,3,5-3TPE-thiophene (10 uM) in THF.

2.7 Infrared Spectra

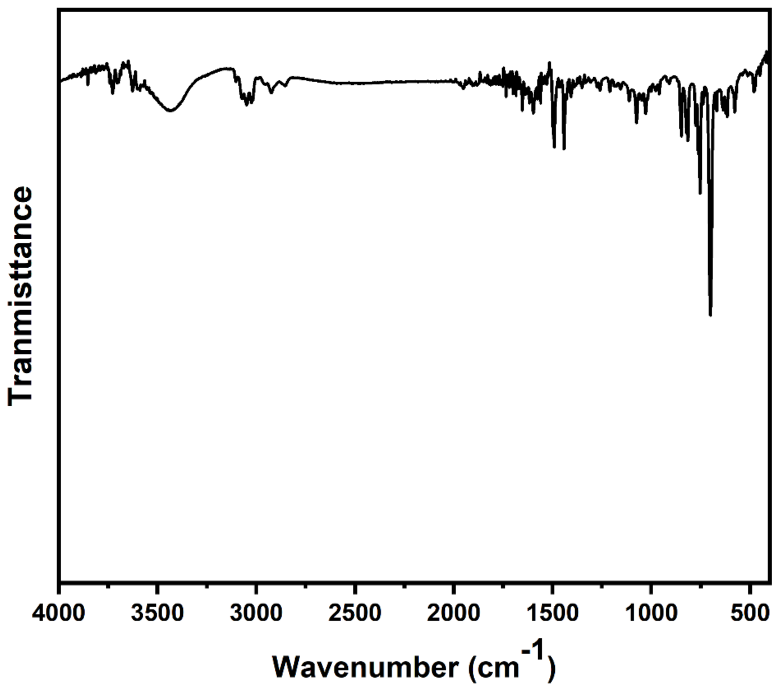

Figure S24. FT-IR spectrum of 2-TPE-thiophene.

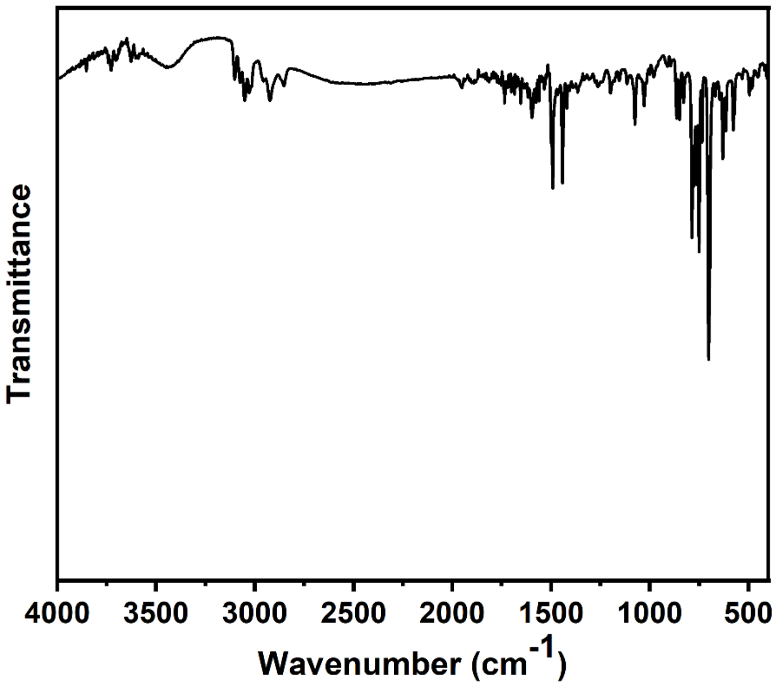

Figure S25. FT-IR spectrum of 3-TPE-thiophene.

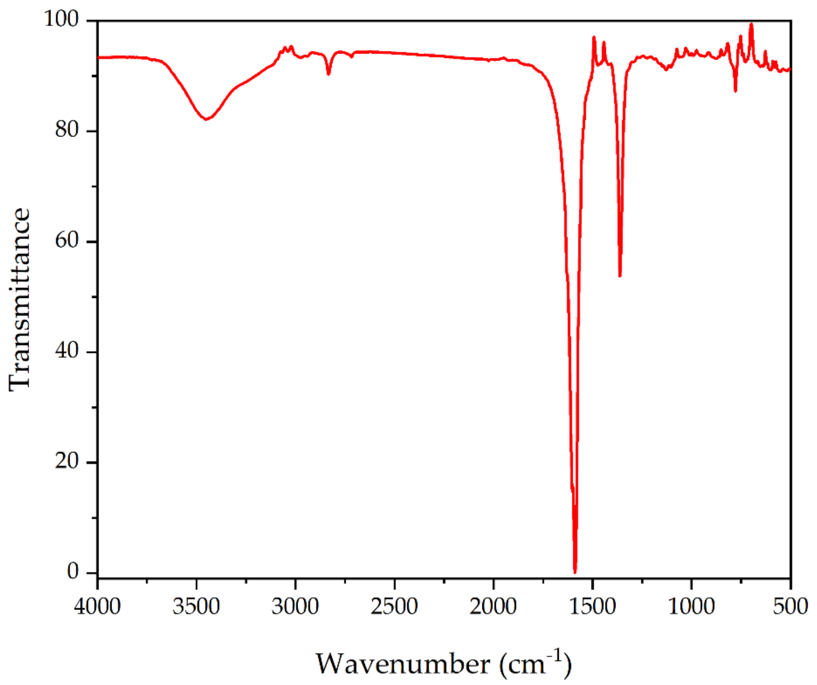

Figure S26. FT-IR spectrum of 2,5-2TPE-thiophene.

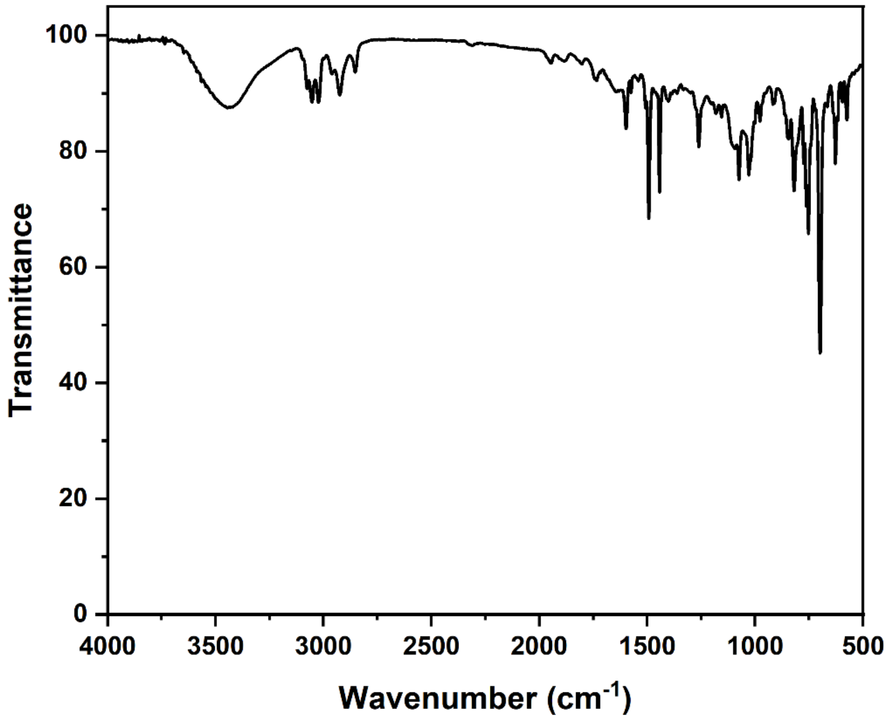

Figure S27. FT-IR spectrum of 2,3,5-3TPE-thiophene.

3 References

1. Frisch, M. J.; Trucks, G. W.; Schlegel, H. B.; Scuseria, G. E.; Robb, M. A.; Cheeseman, J. R.; Scalmani, G.; Barone, V.; Petersson, G. A.; Nakatsuji, H.; Li, X.; Caricato, M.; Marenich, A. V.; Bloino, J.; Janesko, B. G.; Gomperts, R.; Mennucci, B.; Hratchian, H. P.; Ortiz, J. V.; Izmaylov, A. F.; Sonnenberg, J. L.; Williams; Ding, F.; Lipparini, F.; Egidi, F.; Goings, J.; Peng, B.; Petrone, A.; Henderson, T.; Ranasinghe, D.; Zakrzewski, V. G.; Gao, J.; Rega, N.; Zheng, G.; Liang, W.; Hada, M.; Ehara, M.; Toyota, K.; Fukuda, R.; Hasegawa, J.; Ishida, M.; Nakajima, T.; Honda, Y.; Kitao, O.; Nakai, H.; Vreven, T.; Throssell, K.; Montgomery Jr., J. A.; Peralta, J. E.; Ogliaro, F.; Bearpark, M. J.; Heyd, J. J.; Brothers, E. N.; Kudin, K. N.; Staroverov, V. N.; Keith, T. A.; Kobayashi, R.; Normand, J.; Raghavachari, K.; Rendell, A. P.; Burant, J. C.; Iyengar, S. S.; Tomasi, J.; Cossi, M.; Millam, J. M.; Klene, M.; Adamo, C.; Cammi, R.; Ochterski, J. W.; Martin, R. L.; Morokuma, K.; Farkas, O.; Foresman, J. B.; Fox, D. J. Gaussian 16 Rev. B.01, Wallingford, CT, 2016.

2 Dennington, R.; Keith, T. A.; Millam, J. M. GaussView, Version 6.1, Semichem Inc.: Shawnee Mission, KS, 2016.

3 Zhu, R.; Pan, Y.; Yu, H.; Huang, C.; Tian, H.; Wang, T.; Xu, J.; Xiao, S. *Chem.—Asian J.* 2023, 18, e202300600.
